# A Systems Biology Study on NFκB Signaling in Primary Mouse Hepatocytes

**DOI:** 10.3389/fphys.2012.00466

**Published:** 2012-12-31

**Authors:** Federico Pinna, Sven Sahle, Katharina Beuke, Michaela Bissinger, Selcan Tuncay, Lorenza A. D’Alessandro, Ralph Gauges, Andreas Raue, Jens Timmer, Ursula Klingmüller, Peter Schirmacher, Ursula Kummer, Kai Breuhahn

**Affiliations:** ^1^Institute of Pathology, University Hospital of HeidelbergHeidelberg, Germany; ^2^Department of Modeling of Biological Processes, Centre for Organismal Studies Heidelberg/BIOQUANT, University of HeidelbergHeidelberg, Germany; ^3^Division of Systems Biology of Signal Transduction, German Cancer Research Center, DKFZ-ZMBH Alliance, BIOQUANTHeidelberg, Germany; ^4^Albstadt Sigmaringen UniversitySigmaringen, Germany; ^5^Department of Data Analysis and Modeling of Dynamic Processes in the Life Sciences, University of FreiburgFreiburg, Germany

**Keywords:** mathematical modeling, p65, IκB, protein degradation, hepatocytes, signaling

## Abstract

The cytokine tumor necrosis factor-alpha (TNFα) is one of the key factors during the priming phase of liver regeneration as well as in hepatocarcinogenesis. TNFα activates the nuclear factor κ-light-chain-enhancer of activated B cells (NFκB) signaling pathway and contributes to the conversion of quiescent hepatocytes to activated hepatocytes that are able to proliferate in response to growth factor stimulation. Different mathematical models have been previously established for TNFα/NFκB signaling in the context of tumor cells. Combining these mathematical models with time-resolved measurements of expression and phosphorylation of TNFα/NFκB pathway constituents in primary mouse hepatocytes revealed that an additional phosphorylation step of the NFκB isoform p65 has to be considered in the mathematical model in order to sufficiently describe the dynamics of pathway activation in the primary cells. Also, we addressed the role of basal protein turnover by experimentally measuring the degradation rate of pivotal players in the absence of TNFα and including this information in the model. To elucidate the impact of variations in the protein degradation rates on TNFα/NFκB signaling on the overall dynamic behavior we used global sensitivity analysis that accounts for parameter uncertainties and showed that degradation and translation of p65 had a major impact on the amplitude and the integral of p65 phosphorylation. Finally, our mathematical model of TNFα/NFκB signaling was able to predict the time-course of the complex formation of p65 and of the inhibitor of NFκB (IκB) in primary mouse hepatocytes, which was experimentally verified. Hence, we here present a mathematical model for TNFα/NFκB signaling in primary mouse hepatocytes that provides an important basis to quantitatively disentangle the complex interplay of multiple factors in liver regeneration and tumorigenesis.

## 1 Introduction

Liver regeneration after tissue damage is a tightly regulated spatio-temporal process, which is primarily controlled by specific growth factors and cytokines (Michalopoulos, 2010). It predominantly relies on the fast initiation of hepatocyte proliferation after injury and only few proliferative cycles are necessary to restore the liver mass and function (Papp et al., 2009).

Tumor necrosis factor-alpha (TNFα), which is predominantly secreted from liver-resident macrophages (Kupffer cells), represents one of the earliest stimuli for proper initiation of hepatocytic proliferation (Yamada et al., 1997). The most extensively characterized response activated by TNFα is the highly conserved nuclear factor of κ-light-chain enhancer of activated B cells (NFκB) pathway, which can initiate hepatocytic mitotic cycles after entering the nucleus. After partial hepatectomy (PHx), which is the best experimental model for studying liver regeneration, the level of TNFα increases dramatically within 1 h, leading to fast activation of NFκB signaling (Diehl and Rai, 1996). The pivotal relevance of TNFα-induced signaling is supported by the fact that in type I TNFα receptor-deficient mice, hepatocyte proliferation is strongly reduced and liver regeneration is impaired after PHx (Yamada et al., 1997).

Activation of the NFκB axis involves the formation of homo- or hetero-dimeric transcription factors composed of different subunits: p50 (NFκB1), p52 (NFκB2), p65 (RelA), RelB, and c-Rel (Hayden and Ghosh, 2012). In absence of pathway activation, these dimers are disabled by a family of inhibitory proteins including IκBα, IκBβ, IκBε, and IκBγ. For example, binding of IκBα to hetero-dimeric p65:p50 results in steady-state cytoplasmic complex retention and therefore prevents dimers from binding their respective DNA recognition sites. Upon TNFα stimulation, different regulatory proteins are recruited in close proximity to the TNFα receptor and form the receptor-associated signalosome. Subsequently, IκBα phosphorylation is mediated by a kinase complex composed of IκB kinase-α (IKKα), IκB kinase-β (IKKβ), and IκB kinase-γ (IKKγ or NEMO), followed by proteasomal degradation of inhibitory IκBα as well as release and nuclear translocation of p65:p50 complexes. These nuclear heterodimers induce transcription of genes involved in the initiation of proliferation as well as negative regulation of apoptosis (Hayden and Ghosh, 2012).

The modulation of NFκB signaling results in different expression signatures *in vivo* and *in vitro* (Ashall et al., 2009; Li et al., 2009; Sung et al., 2009). This could be achieved by posttranslational modifications such as alternative phosphorylation of the involved signaling components. Multiple phosphorylation sites have been described for the NFκB subunit p65 (Viatour et al., 2005). Most common is the phosphorylation of p65 at serine 536 that is mediated by IKK-β, Akt, and RSK1 in response to TNFα (Sakurai et al., 1999), and IL1β (Madrid et al., 2001) stimulation and DNA damage (Bohuslav et al., 2004). Other phosphorylation sites and kinases are gaining importance for regulating p65 transcriptional activity.

The protein kinase C, zeta (PKCζ) is capable to phosphorylate free p65 at serine 311 when translocated into the nucleus following TNFα stimulation (Duran et al., 2003). Furthermore another specific p65 phospho-site, serine 276, has been shown to be a target for protein kinase A, c (PKAc), and mitogen-activated protein kinase 14 (MAPK14/MSK1; Vermeulen et al., 2003; Jamaluddin et al., 2007). These kinases are of particular interest since they are localized in the nucleus and provide the possibility to modify the transcriptional activity of p65 that translocates to the nucleus subsequent to phosphorylation at serine 536 by IKK-β. However, currently there is not much known regarding their direct involvement in the regulation of canonical NFκB signaling (Joo and Jetten, 2008) and the efficiency of antibodies used for detection are controversially discussed (Spooren et al., 2010).

Another important feature of NFκB signaling is ligand-independent, basal, and ligand-dependent turnover of the signaling components. Basal degradation of IκBα has been reported (Krappmann et al., 1996; Pando and Verma, 2000) suggesting that two different IκBα pools, p65-bound and free unbound, exist. In both cases, IKK-β-mediated IκBα phosphorylation was not necessary for basal protein degradation. However, the pool of free IκBα represents only 15% of the total protein amount (Rice, 1993) and thus specific mechanisms are required to explain basal degradation of free and p65-bound IκBα (Mathes et al., 2008). Combining genetic tools and mathematical models Mathes et al showed that free IκBα degradation is independent of its phosphorylation or ubiquitination but mainly mediated by a specific C-terminal sequence (PEST) able to fine-tune turnover of free IκBα protein. On the other hand, different from the rather instable free IκBα, p65 is relatively stable and upon complex formation with IκBα protects IκBα from fast degradation. IKK-β-mediated phosphorylation of p65-bound IκBα in response to TNFα stimulation leads to the release of the complex unmasking the PEST sequence and results in an efficient recognition and fast degradation by the proteasome. Thus, basal and ligand-dependent turnover operate on different time scales. Their specific contribution to signal amplitude and duration remains to be elucidated.

In the past, the dynamic behavior of NFκB has been intensively analyzed in different cancer cells and immortalized fibroblasts (Cheong et al., 2006; Ashall et al., 2009). Particularly, time-resolved phosphorylation analysis of pathway constituents (including p65 and IκBα) followed by the activation of target genes involved in feedback regulation (e.g., IκBα, A20) leads to the identification of sustained oscillatory behavior in NFκB signaling (Nelson et al., 2004).

In this regard, NFκB signaling has been extensively studied by computational modeling. Carlotti et al. (1999, 2000) presented a first computational study focused on the investigation of the NFκB shuttling, whereas Hoffmann et al. (2002) studied the whole signaling module. The model by Nelson et al. (2004) pointed to the importance of the IκBα transcription rate for the frequency of oscillations and further analysis suggested IκBα and A20 have a major impact on the pathway dynamics (Werner et al., 2008). Another study reported a key role for IκBα and IKKs in oscillations and protein turnover (Ihekwaba et al., 2004; Cheong et al., 2006). Recently, the influence of model parameters on the pathway dynamics has been studied identifying NFκB concentration to be the most decisive parameter for oscillations (Wang et al., 2011).

Only few studies included protein synthesis and degradation rates as relevant parameters for NFκB signaling dynamics (O’Dea et al., 2007). In some cases, IκB turnover is part of the model, but NFκB, e.g., p65 turnover has not been included so far, which is probably due to the relatively stable levels of NFκB. However, it is problematic to predict the influence of stable proteins on systems behavior. O’Dea et al. (2007) measured and modeled the influence of NFκB binding on the degradation rate of IκB species, which was important for their computational predictions. Thus, NFκB seems to protect IκB species from unstimulated degradation. None of the described studies analyzed and modeled NFκB signaling in primary and non-malignantly transformed hepatocytes representing the major cell population of the liver involved in regeneration and tumorigenesis.

Here we present a hepatocyte-specific model for TNFα-induced NFκB signaling that considers basal protein turnover and facilitates the prediction of TNFα-induced complex formation of p65 and IκBα. Since not all parameters of the model are fully identifiable we study an ensemble of 30 different parameter sets that each fit our experimental data. Model validation and conclusions are based on common properties of the ensemble of models.

## 2 Materials and Methods

### 2.1 Experimental: Animal work, isolation of primary murine hepatocytes, and culture conditions

C57Bl/6 mice were obtained from Charles River (Wilmington, MA, USA) and housed under standard conditions in the DKFZ animal facility. All experiments were performed with the approval of the German Regional Council of Baden-Wuerttemberg (Karlsruhe, Germany) and in accordance with the institutional regulations. Isolation of hepatocytes from mice has previously been described (Castoldi et al., 2011). In brief, six to twelve weeks old animals were anesthetized using 10% ketamine hydrochloride (5/100 mg body weight) and 2% xylazine hydrochloride (1/100 mg body weight), and perfused with HANKS solution supplemented with 0.3 mg/ml collagenase CLSII and 5 mM CaCl_2_. After liver removal, hepatocytes were plated on collagen-coated culture dishes (1 million cells/6 cm BD; Biocoat; Horsham, PA, USA) and cultured with adhesion medium (Williams’ medium E (Biochrom, Berlin, Germany), 10% FCS, 0.1% insulin, 100 nM dexamethasone, 2 mM l-glutamine, and 1% penicillin/streptomycin) at 37°C for 4 h. After attachment, cells were washed with PBS (GIBCO Life Technologies, Darmstadt, Germany) and incubated over-night with pre-starvation medium (Williams’ medium E, 100 nM dexamethasone, 2 mM l-glutamine, and 1% penicillin/streptomycin). Before TNFα stimulation, cells were incubated in starvation medium (Williams medium E and 1% penicillin/streptomycin) for 5 h. Dynamic stimulation of NFκB signaling was achieved by administration of recombinant murine TNFα (10 ng/ml; R&D Systems, Minneapolis, MN, USA) in starvation medium. At different time-points after TNFα treatment (5, 10, 20, 40, 60, 120, 180, 240 min), medium has been removed and cells were washed with PBS immediately before mRNA and protein isolation.

#### 2.1.1 mRNA preparation and real-time PCR

For isolation of total mRNA from hepatocytes the NucleoSpin RNA II kit has been used (Macherey-Nagel Düren, Germany). cDNA synthesis (RevertAid H minus, Fermentas, St. Leon-Rot, Germany) and semiquantitative real-time PCR (Abgene/Thermo Fisher, Epsom, UK) have been performed using SYBR Green ROX Mix (Thermo Scientific, Ulm, Germany) according to the manufacturers’ protocol. The following primers were used in this study: IkBα forward: 5′-CCT GGC CAT CGT GGA GCA CT-3′, IkBα reverse: 5′-AGT AGC CTT GGT AGG TTA CC-3′; tubulin forward: 5′-TCA CTG TGC CTG AAC TTA CC-3′; tubulin reverse: 5′-GGA ACA TAG CCG TAA ACT GC-3′ (Thermo Scientific, Ulm, Germany). Cycling program: 95°C/15 min followed by 40 cycles 95°C/15 s and 60°C/1 min. Melting curve: 95°C/15 s, 60°C/30 s, 95°C/15 s.

#### 2.1.2 Protein preparation, immunoprecipitation, and immunoblotting

Total protein fractions were collected at the indicated time-points using the Cell Lysis Buffer (Cell Signalling Technology, Frankfurt am M., Germany) supplemented with Protease Inhibitor Mix G (Serva, Heidelberg, Germany). After sonication (3 times for 20 s) and protein quantification (NanoDrop, Thermo Scientific), 80 μg/lane of total protein were loaded on a denaturing 10% PAA/SDS gel.

To determine time-dependent basal protein degradation of specific NFκB pathway constituents, cells were treated with cycloheximide (50 μg/ml). Proteins were isolated at indicated time-points.

For p65: IκB-α co-immunoprecipitation, proteins were isolated using NP-40 buffer (50 mM Tris-HCl, 150 mM NaCl, 1% NP-40). Three micrograms of p65-specific antibody (clone F6, Santa Cruz Biotechnology, Heidelberg, Germany) and 40 μl G PLUS-Agarose (Santa Cruz Biotechnology) were mixed with 1.5 mg of total protein and incubated over-night. After centrifugation, the pellet was washed three times with HNTG buffer (20 mM Hepes pH 7.5, 150 mM NaCl, 0.1% Triton X-100, and 10% glycerol). Subsequently, the pellet was diluted in 1x sample buffer (100 mM Tris-HCl pH 6.8, 5% glycerol, 0.005% BBP, 5% SDS, 5% β-mercaptoethanol) and incubated at 95°C for 6 min. Pellet was pulled down and supernatant was loaded on a 10% PAA/SDS gel.

After protein transfer, membranes were blocked in 1x TBS containing 0.1% Tween and 5% BSA (Serva, Heidelberg, Germany) or 5% milk for 30 min. The respective primary antibodies were incubated over-night at 4°C. After washing with TBST, membranes were incubated with specific secondary antibody (1 h at RT) and signal detection was performed by ECL (Western Lightning Plus-ECL, Perkin Elmer). Signals were digitally documented using the Fluorchem FC device (Alpha Innotech/Biozym, Hess. Oldendorf, Germany).

Primary antibodies used in this study were: anti-p65 (clone A, dilution for WB: 1:200, Santa Cruz Biotechnology), anti-p65 (clone F6; dilution for IP: 1:25, Santa Cruz Biotechnology), anti-phospho-p65 (Ser536, dilution for WB: 1:500, Santa Cruz Biotechnology), anti-IκB-α (#9242, dilution: 1:750, Cell Signalling Technology), anti-phospho-IκBα (Ser32, clone: 14D4, dilution: 1:400, Cell Signalling Technology), and anti-actin (clone: C4, dilution: 1:2000, MP Biomedical, Germany). Secondary antibodies were: anti-rabbit IgG HRP-linked antibody (dilution: 1:2000, Cell Signalling Technology) and anti-mouse IgG (dilution: 1:2000, Cell Signalling Technology).

#### 2.1.3 Data acquisition and processing

Every experiment was replicated at least eight times and raw data have been used for modeling purposes. For every protein, four digital pictures at different exposure times were taken and signals were quantified using the image analysis software Quantity-One (Biorad, Munich, Germany). Each numerical data point was normalized to respective actin amounts, which served as loading control for the samples. For comparability between different experiments, all data points were further normalized to the appropriate control on each blot (hepatocytes without TNFα stimulation) to a fixed value (c = 100 arbitrary units).

### 2.2 Computational

A system of ordinary differential equations (ODEs) was set up with the software COPASI (Hoops et al., 2006) and integrated using the LSODA integrator as implemented in this software (Petzold, 1983). Sensitivity analysis was employed to determine the influence of certain parameters on systems outcomes, e.g., amplitudes as described in the text. The sensitivity analysis was also used as implemented in COPASI. Parameter fitting was performed using the particle swarm algorithm (Kennedy and Eberhart, 1995), again as implemented in COPASI.

## 3 Results

### 3.1 Initial TNFα/NFκB signaling model

**Figure 1:**
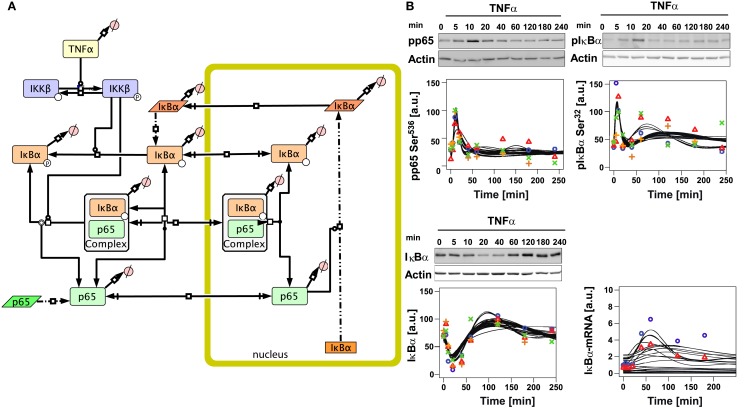
**Initial mathematical model of TNFα/NFκB signaling in hepatocytes**. **(A)** Schematic representation of the initial model created with the software CellDesigner4.2 indicating the considered components and reactions (Funahashi et al., 2008). Boxes with round corners symbolize proteins, straight boxes indicate DNA, and parallelograms represent RNA. **(B)** Time-course of TNFα-induced signaling and model trajectories. 10^6^ primary mouse hepatocytes were stimulated with 10 ng/ml TNFα for the indicated time. From total cellular lysates phosphorylation (pphodpho-serine 536 p65 and phospho-serine 32 IκBα) and total protein (IκBα) were examined by immunoblotting. Equal loading was confirmed by reprobing of the immunoblots with an anti-actin antibody. Quantification was performed using chemiluminescence, detection by the Fluorchem FC device and the image analysis software Quantity-One. Representative immunoblots are indicated above each panel. Quantifications from four independent preparations of primary mouse hepatocytes are indicated by symbols. The time-course of IκBα mRNA induction was determined by real-time PCR. Representative results for two independent experiments are depicted. See Tables S11 and S12 in Supplementary Material for exact values of the experimental data. Black lines indicate model trajectories for 30 parameter sets, fitting and simulation was done with the software COPASI. The concentrations are given in arbitarty units (a. u.).

In order to investigate hepatocyte-specific behavior of NFκB signaling, we developed an initial model based on the most important NFκB pathway constituents reported in the literature for hepatocytes (Wullaert et al., 2007; Figure 1A). The involved processes were modeled similar to published models (O’Dea et al., 2007; Ashall et al., 2009). All processes upstream of the IκB kinase (IKKβ) were lumped in the model assuming that they happen very fast (Delhase et al., 1999). Phosphorylated IKKβ is active and phosphorylates free and p65-bound IκBα. Phosphorylated IκBα dissociates from p65, and is rapidly degraded (Ghosh et al., 1998). Free p65 is imported to the nucleus where it induces transcription of different target genes such as IκBα and A20 (Scott et al., 1993; Tian et al., 2005). In our initial model we introduced stimulated and basal transcription, translation, nuclear mRNA export, and degradation of IκBα as well as IκBα-mRNA. Newly synthesized IκBα can be phosphorylated and degraded or it can reenter the nucleus where it forms a complex with p65 which is then exported to the cytoplasm (Arenzana-Seisdedos et al., 1995). Furthermore, p65 turnover was included in form of translation and degradation reactions while assuming a constant concentration of the corresponding mRNA. Most reactions were modeled as reversible reactions with mass action kinetics, while phosphorylation of complexed and free IκBα was modeled by a Michaelis-Menten kinetics with IKKβ being the catalytic enzyme in this term. The included protein synthesis and degradation for p65 and IκBα, and the reversible kinetics as well as a basal IKKβ phosphorylation led to basal levels of all species with exception of the ligand TNFα. We assumed the model to be in a steady-state before administration of TNFα. For more details on the reactions and the parameters see Tables S1, S3, S5–S7 in Supplementary Material.

### 3.2 Parametrization of the model based on experimental data

To parametrize the initial model of TNFα-induced NFκB signaling, quantitative time-courses of NFκB pathway constituents were measured after TNFα treatment of primary murine hepatocytes. Since it has been shown that TNFα operates very early during liver regeneration (Fausto et al., 2006), we investigated the system for 4 h after TNFα stimulation and measured concentrations and/or activities using narrow time-point sampling, especially in the beginning of this time frame.

We detected the phosphorylation of IκBα and p65 by immunoblotting using total cellular lysates isolated at different time-points after TNFα stimulation. Rather constant levels of total p65 during the time-course were observed (data not shown). However, TNFα stimulation resulted in a rapid increase of phosphorylated p65 after 10 min, followed by a strong decrease until the original level was reached after approximately 40 min (Figure 1B). IκBα was phosphorylated approximately with the same dynamics as p65; however, slightly faster and with a second peak of activation around 60 min, which lasted until 120 min after stimulation (Figure 1B).

In line with the notion that phosphorylation of IκBα is associated with K48-linked polyubiquitination and subsequent degradation, a rapid reduction of the IκBα protein level from 10 to 40 min after TNFα treatment and fast recovery after 60 min until the end of the experiment were detected (Figure 1B). To determine to what extent the p65 activity triggered IκBα gene transcription, real-time PCR analysis was performed revealing increased IκBα mRNA levels starting from 20 min with a maximum peak level around 60 min after stimulation. These experimental data were used to perform parameter estimation for the initial model.

**Figure 2:**
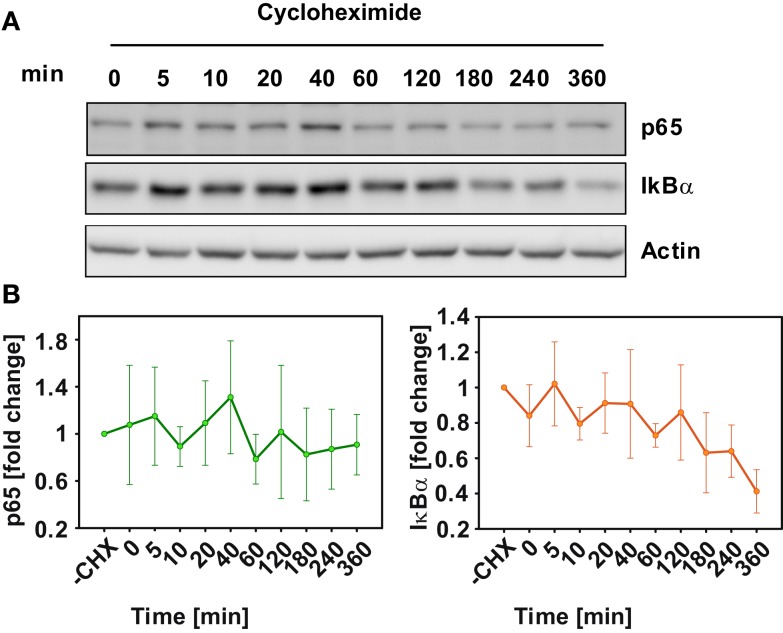
**Basal protein degradation of NFκB pathway components**. In the absence of TNFα, primary mouse hepatocytes were treated with 50 μg/ml cyloheximide and were lyzed at the indicated time-points. **(A)** Representative immunoblot of three independent experiments is shown for IκB and p65. **(B)** Median values and standard deviation of measured p65 and IκBα from three independent experiments.

To further constrain the parameters of the model, basal turnover of the examined signaling components was measured. For this primary mouse hepatocytes were pretreated with the inhibitor of protein synthesis cycloheximide (CHX) and time-courses up to 360 min without TNFα administration were measured for p65 and IκBα by immunoblotting (Figure 2A). The results revealed high p65 stability throughout the entire experimental time frame, indicating little or no p65 turnover. On the other hand, IκBα showed a 40% degradation within 6 h after CHX treatment (Figure 2B). Therefore, a constraint was introduced for parameter estimation to only consider parameter sets that ensured a stable level of p65. Individual parameter values for the degradation of p65 and IκBα in their different binding states could not be derived from the measurements of total p65 and IκBα concentrations. Instead, parameters of p65 and IκBα degradation were constrained in the parameter fitting procedure such that the resulting overall degradation rates were close to the measured values.

The initial model was able to fit most of the data well. The fitting, which was computationally very time consuming, was repeated 60 times with random start values, the 30 best fits were selected for further analysis. However, a more detailed inspection of the resulting model trajectories revealed that simultaneous fitting of phosphorylated p65, IκBα mRNA, and IκBα time-courses was not sufficiently possible with this preliminary model. More specifically, considering the time-courses of phosphorylated p65, phosphorylated IκBα, and total IκBα revealed two distinct clusters of parameter sets, none of which could capture all the features of the observed data. In the first cluster of parameter sets only the initial fast increase of phosphorylated p65 was captured, the following equally fast decrease however was predicted to be slower than the experimental data would suggest; furthermore, IκBα levels in the model did not decrease as strong as the experimental data would suggest. In the second group of parameter sets the dynamics of phosphorylated p65 were captured better, while total IκBα levels went into a too strong overshoot above the steady-state concentration at approximately 100 min. Also the amplitude of the IκBα mRNA dynamics was significantly smaller than the measurements suggested. These inconsistencies are due to a tight coupling of free p65 and IκBα levels in the initial model and cannot be resolved without changes to the model structure.

### 3.3 Extended model including additional p65 phosphorylation

**Figure 3:**
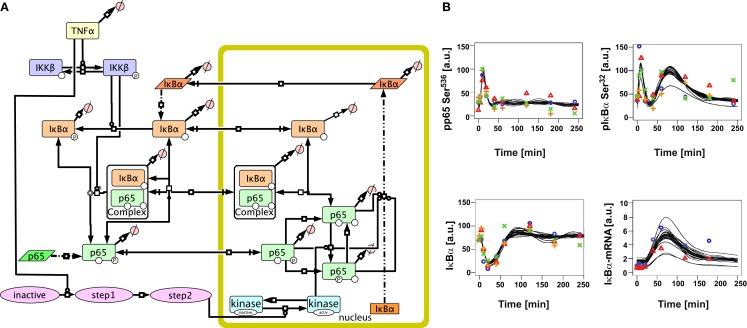
**Extended mathematical model of TNFα/NFκB signaling in hepatocytes**. **(A)** Schematic representation of the extended model created with the software CellDesigner4.2 indicating the additional steps added to the initial model. **(B)** Time-course of TNFα-induced signaling and model trajectories of the extended model. Experimental data for phospho-65, phospho-IκBα, IκBα, and IκBα mRNA as shown in Figure 1 is depicted by symbols. The predicted model trajectories for 30 parameter sets are indicated by black lines. The concentrations are given in arbitarty units (a. u.).

To resolve these discrepancies and improve the description of the experimentally observed induction of IκBα mRNA and subsequent protein levels, we introduced into the initial model reactions that could modify the transcriptional activity of p65 in the nucleus. Since besides phosphorylation at serine 536 additional phosphorylation of p65 by nuclear kinases has been proposed, which could modify target gene expression including IκBα, we considered the activation of a nuclear kinase. The activation of the nuclear kinase was modeled generically by three reaction steps with mass action kinetics to account for a delay in activation, avoiding specific aspects of the upstream activation of the nuclear kinase (Figure 3A, Tables S2, S4 in Supplementary Material).

This extended model was now able to quantitatively fit all experimental data (Figure 3B). In order to analyze the uniqueness of the fit and thus parameter identifiability (Raue et al., 2009), the fitting procedure was repeated 30 times with random start values resulting in 30 parameter sets fitting the experimental data well. Most of the parameters were not identifiable as depicted in Tables S8–S10 in Supplementary Material. However, the kinetic constants for IKKβ dephosphorylation and IκBα basal transcription could be narrowed down to a large extent.

### 3.4 Impact of protein turnover on the dynamics of TNFα/NFκB signaling

The role of protein turnover has been neglected in most of the studies involving NFκB signaling. However, IκBα degradation might be a critical step because its protein levels immediately decreased after activation of the system (IκBα phosphorylation). In addition, the system’s main activated effector, p65, is in turn able to increase IκBα protein levels by promoting its transcription, thus establishing a negative feedback loop in the system.

**Figure 4:**
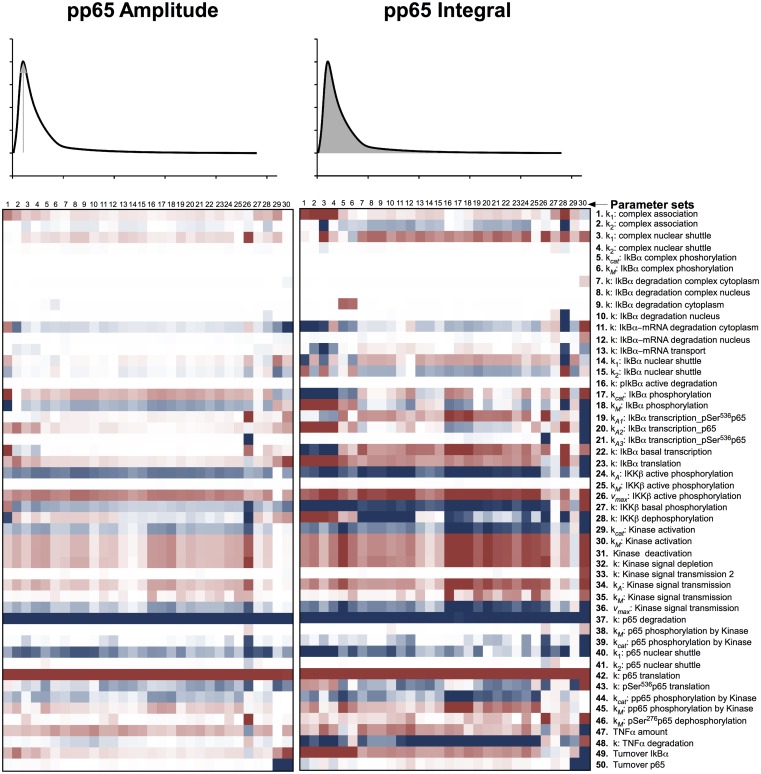
**Impact of parameter changes on amplitude and integral of phospho-p65**. For each of 30 parameter sets the sensitivities with respect to every model parameter are shown in a heat map. Blue shades denote negative, red shades positive sensitivity coefficients. Color shading is scaled from −1.0 to +1.0. The parameter sets were clustered hierarchically according to the shown sensitivities, however, this is merely to improve visual survey and there is no direct correlation to the similarity of parameters. The clustering was preformed with the help of the software Cluster 3.0 (de Hoon et al., 2004).

**Figure A1:**
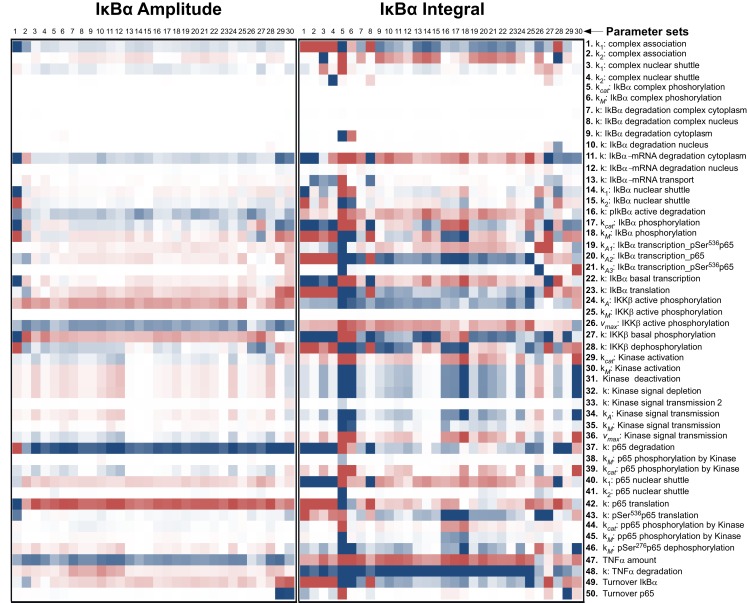
**Impact of parameter changes on amplitude and integral of IκBα**. For each of 30 parameter sets the sensitivities with respect to every model parameter are shown in a heat map. Blue shades denote negative, red shades positive sensitivity coefficients. Color shading is scaled from −1.0 to +1.0. The parameter sets were clustered hierarchically according to the shown sensitivities, however, this is merely to improve visual survay and there is no direct correlation to the similarity of parameters. The clustering was preformed with the help of the software Cluster 3.0 (Ashall et al., 2009).

**Figure A2:**
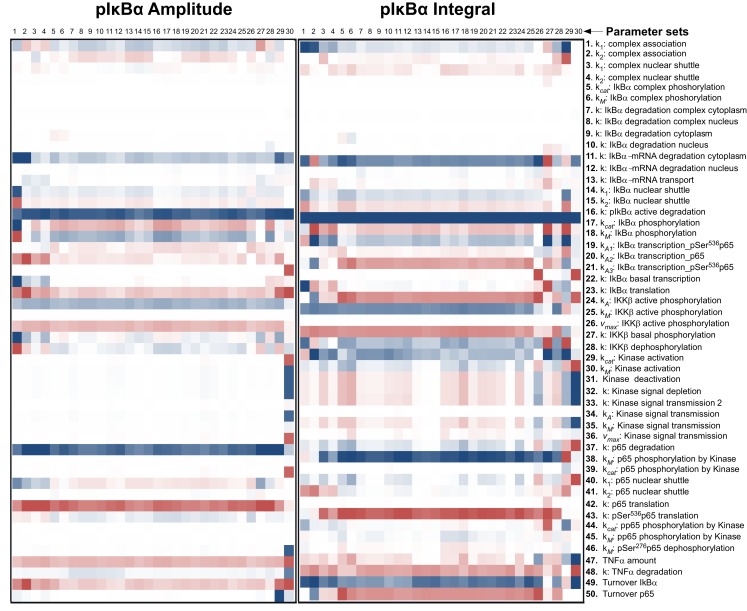
**Impact of parameter changes on amplitude and integral of phospho-IκBα**. For each of 30 parameter sets the sensitivities with respect to every model parameter are shown in a heat map. Blue shades denote negative, red shades positive sensitivity coefficients. Color shading is scaled from −1.0 to +1.0. The parameter sets were clustered hierarchically according to the shown sensitivities, however, this is merely to improve visual survey and there is no direct correlation to the similarity of parameters. The clustering was preformed with the help of the software Cluster 3.0 (Ashall et al., 2009).

**Figure A3:**
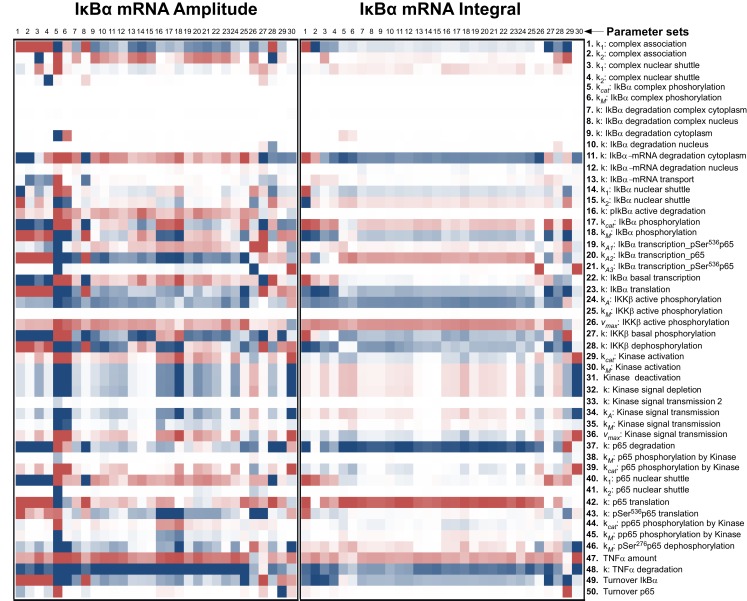
**Impact of parameter changes on amplitude and integral of IκBα mRNA**. For each of 30 parameter sets the sensitivities with respect to every model parameter are shown in a heat map. Blue shades denote negative, red shades positive sensitivity coefficients. Color shading is scaled from −1.0 to +1.0. The parameter sets were clustered hierarchically according to the shown sensitivities, however, this is merely to improve visual survey and there is no direct correlation to the similarity of parameters. The clustering was preformed with the help of the software Cluster 3.0 (Ashall et al., 2009).

Therefore, we computationally analyzed the impact of protein degradation and turnover on important signaling readouts such as the amplitude peak of phosphorylated p65 or the integrated response (area under curve) which represent the signal strength. The ensemble of model parametrizations produced by 30 independent fits was analyzed again. Each of these parametrizations was subjected to a sensitivity analysis calculating the impact of specific kinetic parameters and protein turnover on the peak-height (amplitude) as well as integrated response of the signal (concentration of NFκB). The results demonstrated that the properties of these models were surprisingly robust as depicted in Figure 4 (for the respective heat maps of the IκBα, phosphorylated IκBα, and IκBα mRNA amplitude and integral see Figures A1–A3).

These heat maps showed a high consistency within the 30 different fits despite the unidentifiability of most parameters, indicating that these sensitivities were valid for most parameter sets and therefore most likely for physiological parameter sets. Interestingly, few parameter sets, e.g., parameter set 30, showed differences in comparison with most parameter sets. Analyzing the specific behavior of these unique models, revealed a high sensitivity toward many parameters, indicating these systems to be in an instable state with respect to perturbation. Therefore, it can be assumed that these few models, although representing the data well, do not correspond to the physiological states in cells.

When analyzing especially the parameters involved in protein synthesis and degradation, which regulate protein level, it became apparent that among the prominent parameters influencing the integral of the signal were those involved in transferring the TNFα stimulation into phosphorylated p65 liberation in form of IKKβ phosphorylation (parameters 24, 26, 27 in Figure 4) and p65 translation and degradation (parameters 42, 37). Interestingly, changing translation and degradation of p65 proportionally, so that turnover was changed but the protein level stayed constant, showed very little impact (parameter 50). Parameters describing basal IκBα transcription, translation, and turnover (parameters 22, 23, 49) were also influencing the integral drastically, whilst its degradation and active transcription (parameters 7–10, 19–21) showed very little impact.

Among the parameters determining the signal amplitude were degradation and translation of p65 (parameters 37, 42); again, despite its turnover (parameter 49) having very little influence. In general, the amplitude of phosphorylated p65 was controlled by fewer parameters than the integral of phosphorylated p65.

With respect to the amplitudes and integrals of phosphorylated p65, total IκBα, phosphorylated IκBα, and IκBα mRNA, the system typically depended on the p65 translation and degradation (parameters 37, 42), the activated IKKβ phosphorylation (parameters 24, 26), and the IκBα mRNA degradation in the cytoplasm (parameter 11), whilst changing p65 turnover (assuming unchanged steady-state levels of p65, parameter 49) had limited impact.

### 3.5 Validation of the model and prediction of complex formation

**Figure 5:**
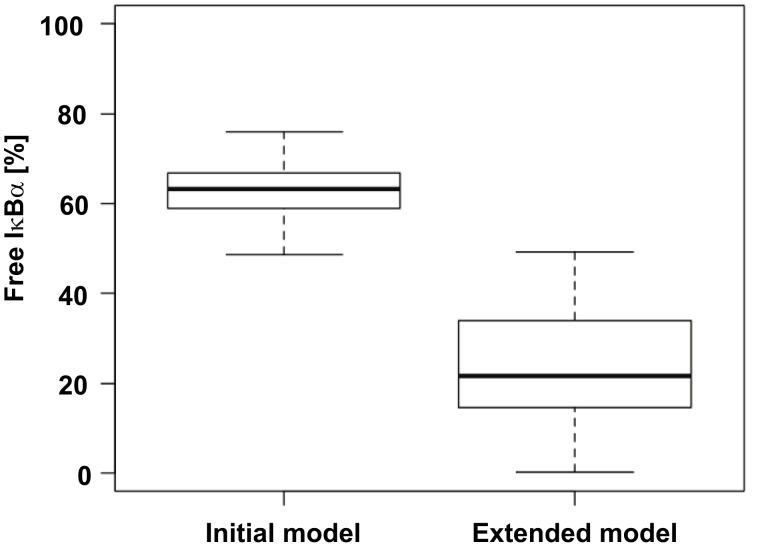
**Comparison of free IκBα predicted by the initial and the extended model**. The level of free, unphosphorylated IκBα for 30 parameter sets of both models was predicted and is depicted as a box plot. According to literature at most 15% of IκBα is unbound of p65 (Rice, 1993).

To assess improvements achieved through the development of the extended model, we compared the ratios of free IκBα before TNFα administration predicted by the initial model and the extended model. In the initial model all parameters sets propose that at least 50% of total IκBα is in an unbound state. On the other hand in the extended model the median lies at 20% (Figure 5) which is well in line with the experimentally observed 15% reported by Rice (1993).

**Figure 6:**
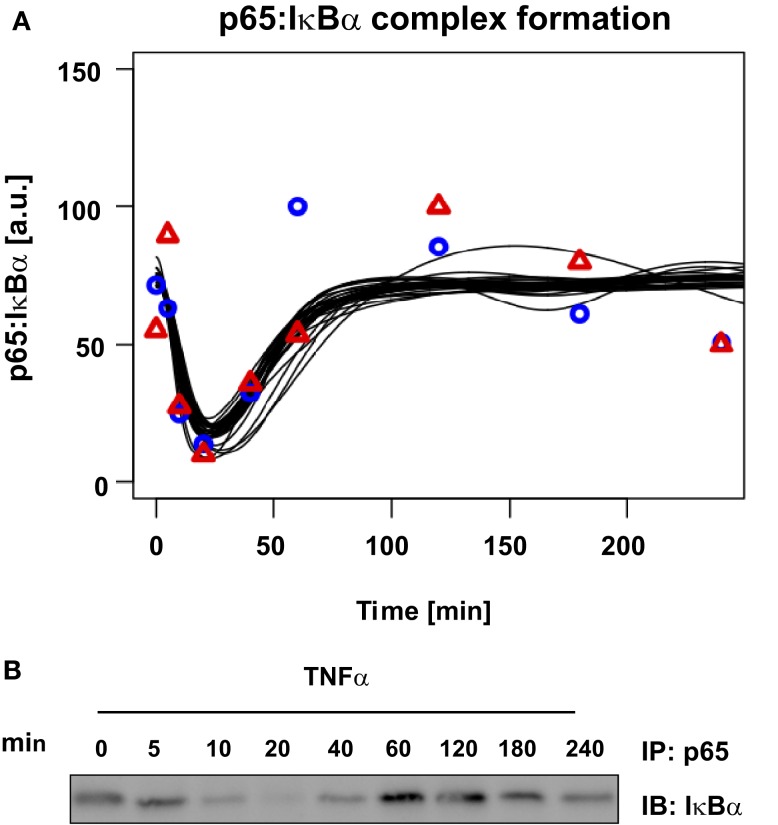
**Model prediction and experimental validation of p65: IκBα complex formation**. About 10^6^ primary mouse hepatocytes were stimulated with 10 ng/ml TNFα for the indicated time. From NP-40 lysates complexes of p65 and IκBα were co-immunoprecipitated (IP) using an anti-p65 antibody and probing the immunoblots (IB) with an anti-IκBα antibody. A representative immunoblot is shown and quantifications for two independent hepatocyte preparations are indicated by symbols. Black lines indicate the time-course predictions from 30 parameter sets.

Finally, we validated our mathematical model of TNFα-induced NFκB signaling in primary mouse hepatocytes by using experimental data not utilized for parametrization of the model. p65:IκBα co-immunoprecipitation was performed to measure complexed p65 concentrations over time (Figure 6B). Since most of the parameter values were not identifiable, we created a model ensemble by conducting the parameter fitting 30 times. All 30 fits, representing different parameter sets all fitting the experimental data, were used to predict the observed experimental co-immunoprecipitation data. For this purpose, the kinetic parameters were kept unchanged; only the scaling factors were refitted to the validation data (Figure 6A).

The experimental results showed the initial level of p65:IκBα complexes strongly decreased 5 to 10 min after stimulation with TNFα. During this time frame, concentrations of serine 536 phosphorylated p65 increased and IκBα concentration decreased, while after 40 min the IκBα and complex concentrations increased again. Between 60 and 120 min the complexed p65:IκBα reached their initial level, followed by a second minor decrease. The model predicted the full dynamics until the second decrease at approximately 180 min, which was likely due to additional feedback loops in the system.

## 4 Discussion

The involvement of NFκB signaling is critical in the context of liver injury and regeneration as well as inflammation and tumorigenesis (Luedde and Schwabe, 2011). Especially during chronic liver disease, activation of this pathway triggers hepatocyte proliferation and (at later time-points) is eventually supporting tumor development and progression. Hepatocellular carcinoma (HCC) is the third most common cause of cancer-related death worldwide (Breuhahn et al., 2011) and represents a paradigm of inflammation-induced cancer, indicating a strong link between activation of pro-inflammatory pathways such as TNF-induced NFκB signaling and tumorigenesis. However, the impact of NFκB signaling on early phases of hepatocarcinogenesis is highly complex, since this pathway is activated in different cell types including hepatocytes (which represent the major cell population), hepatic stellate cells, and resident immune cells (Kupffer cells) (Amann et al., 2009). In order to understand the impact of this pathway in the earliest stages of liver damage and tumorigenesis, it is of central relevance to generate mathematical models describing its dynamic behavior in normal and non-malignantly transformed cells. These tools represent the basis for further comprehensive systems biology studies, essential for a deeper understanding of liver cancer initiation and progression.

Our initial model for TNFα-induced NFκB signaling in mouse hepatocytes was constructed by applying central aspects of established computational models of NFκB signaling in other organisms or cell types (O’Dea et al., 2007; Ashall et al., 2009). Our goal was a reparametrization of this model using hepatocyte-specific experimental data obtained from comprehensive, quantitative, and time-resolved protein and transcript analyses. However, the model was not able to satisfyingly fit all experimental data simultaneously. Specifically, it was not able to describe the difference in the dynamics of phospho-p65 and free IκBα concentrations during the first hour after administration of TNFα. While the initial rise of phospho-p65 and the corresponding decrease of IκBα occur on a similar fast time scale, phospho-p65 returns to near its steady-state value faster than IκBα. In the initial model, the dephosphorylation of p65 is assumed to coincide with its binding to IκBα implying it cannot happen faster than the regeneration of IκBα. Since this is contrary to our observations, we uncoupled p65 dephosphorylation from its binding to IκBα by introducing a more detailed description of p65 phosphorylation and dephosphorylation including a state in which p65 is not phosphorylated but also not bound to IκBα in the nucleus.

It is well known that p65 can be phosphorylated by a number of different kinases at different residues such as serine 536, which is measured by our immunoblotting approach (Viatour et al., 2005). Among the different kinases that may effect serine 536 phosphorylation, only IKKβ is activated by TNFα-induced signaling (Viatour et al., 2005). This is the process that is implicitly included in our initial model. The extended model contains in addition the phosphorylation of p65 at serine 276 by a nuclear kinase. Our data suggests uncoupling of p65 phosphorylation and its binding to IκBα, which was achieved by implementing an additional phosphorylation and corresponding dephosphorylation process in the model. Adjusted like this, the model can indeed reproduce the observed differences in the dynamics of phospho-p65 and free IκBα. In addition, the revised model also convincingly reproduced the amplitude of the IκBα mRNA measurements, which was not possible using the initial model.

A potential candidate for such a nuclear kinase could be mitogen- and stress-activated protein kinase 1 (MSK1) that has also been shown to be expressed in human liver cells (Deak et al., 1998). However, MSK1 appears to have no direct impact on IκBα expression and the phosphorylation of p65 by MSK1 is controversially discussed (Joo and Jetten, 2008).

The extended model is able to explain features of NFκB signaling in hepatocytes as displayed by our measurements. However, NFκB signaling is known to involve more constituents and regulations than considered in this study. Therefore, other components can be included in future research. Tumor necrosis factor α-induced protein 3 (TNFAIP3/A20) is one considerable protein, involved in a negative loop inhibiting p65 phosphorylation acting upstream of IKK-β (Opipari et al., 1990). This protein, together with IκBα is probably the most important factor in the acquisition of phosphorylated p65 oscillatory behavior critical for transcriptional target fate (Ashall et al., 2009). Another NFκB constituent involved in negative feedback loop with high impact in NFκB signaling oscillations is IκBε. This protein belongs to the IκB inhibitor family of signaling whose activity has been detected later and out of phase if compared with IκBα. This special feature leads IκBε to dampen the effect of IκBα during the last phase of sustained NFκB activity and to mediate the terminations of the signaling in response to transient stimulation (Kearns et al., 2006). From our preliminary data, both protein TNFAIP3/A20 and IκBε present a significant expression in primary hepatocytes following TNFα stimulation and this data will be used for parametrization of further improved models. Moreover signals coming not directly from TNFα but able to fine-tune the system especially in the nucleus should be taken into account.

A vital methodological question remains in the context of this work: What is the justification to claim that the model proposed here is a model for NFκB signaling in mouse hepatocytes? We have consulted literature to make sure all the processes and components included in the model are actually present in mammalian hepatocytes. On the other hand, to our knowledge none of these components or processes is actually exclusive to liver cells or hepatocytes. Basically this leaves the parametrization of the model as being hepatocyte-specific. Parameter estimation was performed with data measured in murine hepatocytes and the presented model was able to fit the data satisfyingly. However, some of the parameters are *unidentifiable* with the available data, i.e., for different values of the parameter the model fits the data equally well. Instead of eliminating the unidentifiabilities – by performing additional experiments and/or by simplifying the model – here we focused on making predictions from the model that are valid for all parametrizations of the model that are compatible with the experimental data. As a pragmatic way to characterize the set of parameter values that let the model fit the data, a heuristic sampling of parameter space was performed, a technique that was recently applied in other studies (Maiwald et al., 2010; Levering et al., 2012; Wegner et al., 2012). The parameter estimation process was repeated 30 times, each time starting with different random parameter values as initial guess, resulting in 30 different sets of parameters that are compatible with the experimental data. This immediately results in a good estimate for the identifiability of the different parameters: the more the parameters differ between the repeated parameter estimation runs the less identifiable they are.

For drawing conclusions from our model or creating predictions for validation we need to make sure that these conclusions or predictions are valid for all or at least most of the 30 parameter sets. Indeed, the model validation described in the *Results* section proved to be conserved for all 30 parameter sets. Similarly the sensitivities that were calculated in order to study the role of protein turnover turned out to be consistent for 25 of the 30 parameter sets. While of course we cannot be absolutely certain from using only a limited number of samples, we can confidently claim that *typically* the model will predict a certain behavior of NFκB signaling in hepatocytes whenever the parameters are chosen so that it fits the data.

While our model shares its basic structure with most relevant NFκB models in literature, it is based on NFκB dynamics following TNFα stimulation in murine primary hepatocytes. Our experimental setup allows us to disturb hepatocytes with different combination of ligands and to compare data with prediction from our model. Different models for different signaling pathways essential for liver homeostasis and response to injury are currently being developed, e.g., within the German Virtual Liver Network (VLN). In this context our model can be a useful tool for comparison and integration of different models. Thus, we have established a mathematical model for TNFα/NFκB signaling in primary mouse hepatocytes that indicated the importance of basal turnover of the pathway components for the dynamic behavior and facilitated the prediction of the kinetics of complex formation in the pathway. Taken together the model provides an important building block to unravel regulation of liver regeneration and the impact of inflammatory responses across the scales.

## Conflict of Interest Statement

The authors declare that the research was conducted in the absence of any commercial or financial relationships that could be construed as a potential conflict of interest.

## Supplementary Material

The Supplementary Material for this article can be found online at http://www.frontiersin.org/Systems_Biology/10.3389/fphys.2012.00466/abstract

Supplementary Table S1 **Ordinary differential equations describing all species in the original model**. As initial concentrations the steady-state concentrations were used.

Supplementary Table S2 **Ordinary differential equations describing all species in the model expanded by p65 phosphorylaiton**.

Supplementary Table S3 **Parameters and fitting boundaries of the parameters of the initial model**.

Supplementary Table S4 **Parameters and fitting boundaries of the parameters of the expanded model**.

Supplementary Table S5 **Objective function value and exact values of all parameters fitted via particle swarm in the parameter estimation starting from random start values for the initial model part I**.

Supplementary Table S6 **Objective function value and exact values of all parameters fitted via particle swarm in the parameter estimation starting from random start values for the initial model part II**.

Supplementary Table S7 **Objective function value and exact values of all parameters fitted via particle swarm in the parameter estimation starting from random start values for the initial model part III**.

Supplementary Table S8 **Objective function value and exact values of all parameters fitted via particle swarm in the parameter estimation starting from random start values for the expanded model part I**.

Supplementary Table S9 **Objective function value and exact values of all parameters fitted via particle swarm in the parameter estimation starting from random start values for the expanded model part II**.

Supplementary Table S10 **Objective function value and exact values of all parameters fitted via particle swarm in the parameter estimation starting from random start values for the expanded model part III**.

Supplementary Table S11 **Relative protein concentrations over time determined from immuno blotting**.

Supplementary Table S12 **Relative mRNA concentrations over time determined from quantitative reverse transcription polymerase chain reaction**.

## Research Topic:

From structural to molecular systems biology: experimental and computational approaches to unravel mechanisms of kinase activity regulation in cancer and neurodegeneration

## Topic Editor:

Matteo Barberis, Humboldt University Berlin, Germany; Max Planck Institute for Molecular Genetics, Berlin, Germany

## References

[B1] Amann T. Bataille F. Spruss T. Mühlbauer M. Gäbele E. Schölmerich J. (2009). Activated hepatic stellate cells promote tumorigenicity of hepatocellular carcinoma. Cancer Sci. 100, 646–653 10.1111/j.1349-7006.2009.01087.x 19175606 PMC11158780

[B2] Arenzana-Seisdedos F. Thompson J. Rodriguez M. S. Bachelerie F. Thoma D. Hay R. T. (1995). Inducible nuclear expression of newly synthesized Iκ Bα negatively regulates dna-binding and transcriptional activities of NFκ-B. Mol. Cell. Biol. 15, 2689–2696 7739549 10.1128/mcb.15.5.2689 PMC230499

[B3] Ashall L. Horton C. A. Nelson D. E. Paszek P. Harper C. V. Sillitoe K. (2009). Pulsatile stimulation determines timing and specificity of NFκ-B-dependent transcription. Science 324, 242–246 10.1126/science.1164860 19359585 PMC2785900

[B4] Bohuslav J. Chen L.-F. Kwon H. Mu Y. Greene W. C. (2004). p53 induces NFκ-B activation by an Iκ B kinase-independent mechanism involving phosphorylation of p65 by ribosomal S6 kinase 1. J. Biol. Chem. 279, 26115–26125 10.1074/jbc.M313509200 15073170

[B5] Breuhahn K. Gores G. Schirmacher P. (2011). Strategies for hepatocellular carcinoma therapy and diagnostics: lessons learned from high throughput and profiling approaches. Hepatology 53, 2112–2121 10.1002/hep.24313 21433041

[B6] Carlotti F. Chapman R. Dower S. K. Qwarnstrom E. E. (1999). Activation of nuclear factor κ (B in single living cells. dependence of nuclear translocation and anti-apoptotic function on egfprela concentration. J. Biol. Chem. 274, 37941–37949 10.1074/jbc.274.53.37941 10608861

[B7] Carlotti F. Dower S. K. Qwarnstrom E. E. (2000). Dynamic shuttling of nuclear factor κB between the nucleus and cytoplasm as a consequence of inhibitor dissociation. J. Biol. Chem. 275, 41028–41034 10.1074/jbc.M006179200 11024020

[B8] Castoldi M. Vujic Spasic M. Altamura S. Elmén J. Lindow M. Kiss J. (2011). The liver-specific microrna mir-122 controls systemic iron homeostasis in mice. J. Clin. Invest. 121, 1386–1396 10.1172/JCI44883 21364282 PMC3069782

[B9] Cheong R. Bergmann A. Werner S. L. Regal J. Hoffmann A. Levchenko A. (2006). Transient IκB kinase activity mediates temporal NFκ-B dynamics in response to a wide range of tumor necrosis factorα-doses. J. Biol. Chem. 281, 2945–2950 10.1074/jbc.M510085200 16321974

[B10] de Hoon M. Imoto S. Nolan J. Miyano S. (2004). Open source clustering software. Bioinformatics 20, 1453–1454 10.1093/bioinformatics/bth078 14871861

[B11] Deak M. Clifton A. D. Lucocq L. M. Alessi D. R. (1998). Mitogen- and stress-activated protein kinase-1 (MSK1) is directly activated by MAPK and SAPK2/p38, and may mediate activation of CREB. EMBO J. 17, 4426–4441 10.1093/emboj/17.15.4426 9687510 PMC1170775

[B12] Delhase M. Hayakawa M. Chen Y. Karin M. (1999). Positive and negative regulation of IκB kinase activity through IKKβ subunit phosphorylation. Science 284, 309–313 10.1126/science.284.5412.309 10195894

[B13] Diehl A. M. Rai R. (1996). Review: regulation of liver regeneration by pro-inflammatory cytokines. J. Gastroenterol. Hepatol. 11, 466–470 10.1111/j.1440-1746.1996.tb00292.x 8743919

[B14] Duran A. Diaz-Meco M. T. Moscat J. (2003). Essential role of RelA Ser311 phosphorylation by γPKC in NFκ-B transcriptional activation. EMBO J. 22, 3910–3918 10.1093/emboj/cdg370 12881425 PMC169043

[B15] Fausto N. Campbell J. S. Riehle K. J. (2006). Liver regeneration. Hepatology 43(Suppl. 1), S45–S53 10.1002/hep.20969 16447274

[B16] Funahashi A. Matsuoka Y. Jouraku A. Morohashi M. Kikuchi N. Kitano H. (2008). CellDesigner 3.5: a versatile modeling tool for biochemical networks. Proc. IEEE 96, 1254–1265 10.1109/JPROC.2008.925458

[B17] Ghosh S. May M. J. Kopp E. B. (1998). NFκ-B and Rel proteins: evolutionarily conserved mediators of immune responses. Annu. Rev. Immunol. 16, 225–260 10.1146/annurev.immunol.16.1.225 9597130

[B18] Hayden M. S. Ghosh S. (2012). NFκ-B, the first quarter-century: remarkable progress and outstanding questions. Genes Dev. 26, 203–234 10.1101/gad.183434.111 22302935 PMC3278889

[B19] Hoffmann A. Levchenko A. Scott M. L. Baltimore D. (2002). The Iκ B-NFκ-B signaling module: temporal control and selective gene activation. Science 298, 1241–1245 10.1126/science.1071914 12424381

[B20] Hoops S. Sahle S. Gauges R. Lee C. Pahle J. Simus N. (2006). COPASI–a complex pathway simulator. Bioinformatics 22, 3067–3074 10.1093/bioinformatics/btl485 17032683

[B21] Ihekwaba A. E. C. Broomhead D. S. Grimley R. L. Benson N. Kell D. B. (2004). Sensitivity analysis of parameters controlling oscillatory signalling in the NFκ-B pathway: the roles of ikk and Iκ Bα. Syst. Biol. (Stevenage) 1, 93–103 10.1049/sb:20045009 17052119

[B22] Jamaluddin M. Wang S. Boldogh I. Tian B. Brasier A. R. (2007). TNFα-induced NFκ-B/RelA Ser(276) phosphorylation and enhanceosome formation is mediated by an ROS-dependent PKAc pathway. Cell. Signal. 19, 1419–1433 10.1016/j.cellsig.2007.01.020 17317104

[B23] Joo J. H. Jetten A. M. (2008). NFκ-B-dependent transcriptional activation in lung carcinoma cells by farnesol involves p65/RelA(Ser276) phosphorylation via the MEK-MSK1 signaling pathway. J. Biol. Chem. 283, 16391–16399 10.1074/jbc.M800945200 18424438 PMC2423266

[B24] Kearns J. D. Basak S. Werner S. L. Huang C. S. Hoffmann A. (2006). IκBα provides negative feedback to control NFκ-B oscillations, signaling dynamics, and inflammatory gene expression. J. Cell Biol. 173, 659–664 10.1083/jcb.200510155 16735576 PMC2063883

[B25] Kennedy J. Eberhart R. (1995). Particle swarm optimization. Proc. Int. Jt. Conf. Neural Netw. 4, 1942–1948

[B26] Krappmann D. Wulczyn F. G. Scheidereit C. (1996). Different mechanisms control signal-induced degradation and basal turnover of the NF-kappaB inhibitor IκB alpha in vivo. EMBO J. 15, 6716–6726 8978697 PMC452495

[B27] Levering J. Musters M. W. J. M. Bekker M. Bellomo D. Fiedler T. de Vos W. M. (2012). Role of phosphate in the central metabolism of two lactic acid bacteria—a comparative systems biology approach. FEBS J. 279, 1274–1290 10.1111/j.1742-4658.2012.08523.x 22325620

[B28] Li J. Campbell J. S. Mitchell C. McMahan R. S. Yu X. Riehle K. J. (2009). Relationships between deficits in tissue mass and transcriptional programs after partial hepatectomy in mice. Am. J. Pathol. 175, 947–957 10.2353/ajpath.2009.090354 19700759 PMC2731115

[B29] Luedde T. Schwabe R. F. (2011). NFκ-B in the liver – linking injury, fibrosis and hepatocellular carcinoma. Nat. Rev. Gastroenterol. Hepatol. 8, 108–118 10.1038/nrgastro.2010.213 21293511 PMC3295539

[B30] Madrid L. V. Mayo M. W. Reuther J. Y. Baldwin A. S. (2001). Akt stimulates the transactivation potential of the RelA/p65 Subunit of NF-kappa B through utilization of the Iκ (B kinase and activation of the mitogen-activated protein kinase p38. J. Biol. Chem. 276, 18934–18940 10.1074/jbc.M101103200 11259436

[B31] Maiwald T. Schneider A. Busch H. Sahle S. Gretz N. Weiss T. S. (2010). Combining theoretical analysis and experimental data generation reveals IRF9 as a crucial factor for accelerating interferon α-induced early antiviral signalling. FEBS J. 277, 4741–4754 10.1111/j.1742-4658.2010.07880.x 20964804

[B32] Mathes E. O’Dea E. L. Hoffmann A. Ghosh G. (2008). NFκ-B dictates the degradation pathway of IκBα. EMBO J. 27, 1357–1367 10.1038/emboj.2008.91 18401342 PMC2374849

[B33] Michalopoulos G. K. (2010). Liver regeneration after partial hepatectomy: critical analysis of mechanistic dilemmas. Am. J. Pathol. 176, 2–13 10.2353/ajpath.2010.090675 20019184 PMC2797862

[B34] Nelson D. E. Ihekwaba A. E. C. Elliott M. Johnson J. R. Gibney C. A. Foreman B. E. (2004). Oscillations in NFκ-B signaling control the dynamics of gene expression. Science 306, 704–708 10.1126/science.1099962 15499023

[B35] O’Dea E. L. Barken D. Peralta R. Q. Tran K. T. Werner S. L. Kearns J. D. (2007). A homeostatic model of Iκ B metabolism to control constitutive NFκ-B activity. Mol. Syst. Biol. 3, 111 10.1038/msb4100148 17486138 PMC2673708

[B36] Opipari A. W. Boguski M. S. Dixit V. M. (1990). The A20 cDNA induced by tumor necrosis factor α encodes a novel type of zinc finger protein. J. Biol. Chem. 265, 14705–14708 2118515

[B37] Pando M. P. Verma I. M. (2000). Signal-dependent and -independent degradation of free and NFκ-B-bound Iκ Bα. J. Biol. Chem. 275, 21278–21286 10.1074/jbc.M002532200 10801847

[B38] Papp V. Dezsö K. László V. Nagy P. Paku S. (2009). Architectural changes during regenerative and ontogenic liver growth in the rat. Liver Transpl. 15, 177–183 10.1002/lt.21665 19177433

[B39] Petzold L. (1983). Automatic selection of methods for solving stiff and nonstiff systems of ordinary differential equations. SIAM J. Sci. Stat. Comput. 4, 136–148 10.1137/0904010

[B40] Raue A. Kreutz C. Maiwald T. Bachmann J. Schilling M. Klingmüller U. (2009). Structural and practical identifiability analysis of partially observed dynamical models by exploiting the profile likelihood. Bioinformatics 25, 1923–1929 10.1093/bioinformatics/btp358 19505944

[B41] Rice N. R. Ernst M. K. (1993). In vivo control of NFκ-B activation by IκBα. EMBO J. 12, 4685–4695 8223478 10.1002/j.1460-2075.1993.tb06157.x PMC413911

[B42] Sakurai H. Chiba H. Miyoshi H. Sugita T. Toriumi W. (1999). IκB kinases phosphorylate NFκ-B p65 subunit on serine 536 in the transactivation domain. J. Biol. Chem. 274, 30353–30356 10.1074/jbc.274.15.10641 10521409

[B43] Scott M. L. Fujita T. Liou H.-C. Nolan G. P. Baltimore D. (1993). The p65 subunit of NFκ-B regulates IκB by two distinct mechanisms. Genes Dev. 7, 1266–1276 10.1101/gad.7.7a.1266 8319912

[B44] Spooren A. Kolmus K. Vermeulen L. Van Wesemael K. Haegeman G. Gerlo S. (2010). Hunting for serine 276-phosphorylated p65. J. Biomed. Biotechnol. 2010, 275892 10.1155/2010/275892 20204068 PMC2829628

[B45] Sung M.-H. Salvatore L. De Lorenzi R. Indrawan A. Pasparakis M. Hager G. L. (2009). Sustained oscillations of NFκ-B produce distinct genome scanning and gene expression profiles. PLoS ONE 4:e7163 10.1371/journal.pone.0007163 19787057 PMC2747007

[B46] Tian B. Nowak D. E. Jamaluddin M. Wang S. Brasier A. R. (2005). Identification of direct genomic targets downstream of the nuclear factorκ-B transcription factor mediating tumor necrosis factor signaling. J. Biol. Chem. 280, 17435–17448 10.1074/jbc.M502925200 15722553

[B47] Vermeulen L. De Wilde G. Van Damme P. Vanden Berghe W. Haegeman G. (2003). Transcriptional activation of the NFκ-B p65 subunit by mitogen- and stress-activated protein kinase-1 (MSK1). EMBO J. 22, 1313–1324 10.1093/emboj/cdg139 12628924 PMC151081

[B48] Viatour P. Merville M.-P. Bours V. Chariot A. (2005). Phosphorylation of NFκ-B and IκB proteins: implications in cancer and inflammation. Trends Biochem. Sci. 30, 43–52 10.1016/j.tibs.2004.11.009 15653325

[B49] Wang Y. Paszek P. Horton C. Kell D. (2011). Interactions among oscillatory pathways in NFκ-B signalling. BMC Syst. Biol. 5:23 10.1186/1752-0509-5-23 21291535 PMC3050740

[B50] Wegner K. Bachmann A. Schad J.-U. Lucarelli P. Sahle S. Nickel P. (2012). Dynamics and feedback loops in the transforming growth factorβ signaling pathway. Biophys. Chem. 162, 22–34 10.1016/j.bpc.2011.12.003 22284904

[B51] Werner S. L. Kearns J. D. Zadorozhnaya V. Lynch C. O’Dea E. Boldin M. P. (2008). Encoding NFκ-B temporal control in response to tnf: distinct roles for the negative regulators IκBα and a20. Genes Dev. 22, 2093–2101 10.1101/gad.1680708 18676814 PMC2492747

[B52] Wullaert A. van Loo G. Heyninck K. Beyaert R. (2007). Hepatic tumor necrosis factor signaling and nuclear factorκ-b: effects on liver homeostasis and beyond. Endocr. Rev. 28, 365–386 10.1210/er.2006-0031 17431229

[B53] Yamada Y. Kirillova I. Peschon J. J. Fausto N. (1997). Initiation of liver growth by tumor necrosis factor: deficient liver regeneration in mice lacking type i tumor necrosis factor receptor. Proc. Natl. Acad. Sci. U.S.A. 94, 1441–1446 10.1073/pnas.94.20.10862 9037072 PMC19810

