# Supplementary material for: A Systems Biology Study on NFκB Signaling in Primary Mouse Hepatocytes
Source: Front Physiol. 2012 Dec 31;3:466. doi: 10.3389/fphys.2012.00466 (PMC3533138; doi:10.3389/fphys.2012.00466)
Supplement: Supplementary Table S1 — Ordinary differential equations describing all species in the original model. As initial concentrations the steady-state concentrations were used. [file 34120_Kummer_DataSheet1.PDF]

Species	ODE	Comments
TNFα	-$V_{cytoplasm}$ * TNFa degradation k * c(TNFα)	intial value: 10 μM
IKKβ	$V_{cytoplasm} * (\frac{-IKK\beta\ active\ phsophorylation\ v_{max}*c(IKK\beta)*c(TNF\alpha)}{(IKK\beta\ active\ phosphorylation\ k_A*c(IKK\beta)+IKK\beta\ active\ phosphorylation\ k_M*c(IKK\beta)}$<br>$-IKK\beta\ basal\ phosphorylationk * c(IKK\beta)$<br>$+IKK\beta\ dephosphorylation\ k * c(IKK\beta\_p))$	modeled as a moiety<br>with pIKKβ<br>total concentration: 1 μM
IKKβ_p	1-IKKβ	
I$\kappa B\alpha_cyt$	$V_{cytoplasm} * (+I\kappa B\alpha\ translation\ k * c(I\kappa B\alpha\_mRNA\_cyt)$<br>$+p65\ degradation\ k * c(I\kappa B\alpha - p65\_cyt)$<br>$+complex\ association\ k_2 * c(complex\_cyt)$<br>$-I\kappa B\alpha\ deg\ cyt\ k * c(I\kappa B\alpha\_\text{cyt})$<br>$-\frac{I\kappa B\alpha\ phosphorylation\ k_{cat}*c(IKK\beta)*c(I\kappa B\alpha\_cyt)}{I\kappa B\alpha\ phosphorylation\ k_M+c(I\kappa B\alpha\_cyt)}$<br>$-complex\ association\ k_1 * c(p65\_cyt) * c(I\kappa B\alpha\_cyt))$<br>$+I\kappa B\alpha\ import\ k2 * c(I\kappa B\alpha\_nuc)$<br>$-I\kappa B\alpha\ import\ k1 * c(I\kappa B\alpha\_cyt)$	
p65_cyt	$V_{cytoplasm} * (\frac{I\kappa B\alpha\ complex\ phosphorylation\ k_{cat}*c(IKK)*c(I\kappa B\alpha-p65\_cyt)}{I\kappa B\alpha\ complex\ phosphorylation\ k_M+c(I\kappa B\alpha-p65\_cyt)}$<br>$+p65\ translation\ k * c(p65mRNA)$<br>$+I\kappa B\alpha\ deg\ cyt\ k * c(I\kappa B\alpha p65\_cyt)$<br>$-p65\ degradation\ k * c(p65\_cyt)$<br>$+complex\ association\ k_2 * c(I\kappa B\alpha p65\_cyt)$<br>$-complex\ association\ k_1 * c(p65\_cyt) * c(I\kappa B\alpha\_cyt))$<br>$-p65\ nuclear\ shuttle\ k_1 * c(p65\_cyt)$<br>$+p65\ nuclear\ shuttle\ k_2 * c(p65\_nuc)$	
IκBα-p65_cyt	$+complex\ nuclear\ shuttle\ k_1 * c(I\kappa B\alpha - p65\_nuc)$<br>$-complex\ nuclear\ shuttle\ k_2 * c(I\kappa B\alpha - p65\_cyt)$<br>$V_{cytoplasm} * (complex\ association\ k_1 * c(p65\_cyt) * c(I\kappa B\alpha\_cyt)$<br>$-complex\ association\ k_2 * c(I\kappa B\alpha - p65\_cyt)$<br>$-\frac{I\kappa B\alpha\ complex\ phosphorylation\ k_{cat}*c(IKK)*c(I\kappa B\alpha-p65\_cyt)}{I\kappa B\alpha\ complex\ phosphorylation\ k_M+c(I\kappa B\alpha-p65\_cyt)}$<br>$-p65\ deg\ k * c(I\kappa B\alpha - p65\_cyt)$<br>$-I\kappa B\alpha\ deg\ complex\ cyt\ k * c(I\kappa B\alpha - p65\_cyt)$	
pIκBα	$V_{cytoplasm} * \frac{I\kappa B\alpha\ phosphorylation\ k_{cat}*c(IKK)*c(I\kappa B\alpha-p65\_cyt)}{I\kappa B\alpha\ phosphorylation\ k_M+c(I\kappa B\alpha-p65\_cyt)}$<br>$+\frac{I\kappa B\alpha\ complex\ phosphorylation\ k\_cat*c(IKK)*c(I\kappa B\alpha\_cyt)}{I\kappa B\alpha\ complex\ phosphorylation\ k_M+c(I\kappa B\alpha\_cyt)}$	

	$-I\kappa B\alpha\ p\ active\ degradation\ k * c(pI\kappa B\alpha)$	
p65_*nuc*	$V_{nucleus} * I\kappa B\alpha\ deg\ complex\ nuc\ k * c(I\kappa B\alpha - p65\_nuc)$<br>$-p65\ degradation\ k * c(p65\_nuc)$<br>$+k_2 complex\ association * c(I\kappa B\alpha - p65\_nuc)$<br>$-k_1 complex\ association * c(p65\_nuc) * c(I\kappa B\alpha\_nuc))$<br>$+p65\ shuttle\ to\ nucleus\ k1 * c(p65\_cyt)$<br>$-p65\ shuttle\ to\ nucleus\ k2 * c(p65\_nuc)$	
IκBα_*nuc*	$V_{nucleus} * +p65\ degradation\ k * c(I\kappa B\alpha - p65\_nuc)$<br>$+complex\ association\ k_2 * c(I\kappa B\alpha - p65\_nuc)$<br>$-I\kappa B\alpha\ deg\ cyt\ k * c(I\kappa B\alpha\_\mathrm{cyt})$<br>$-complex\ association\ k_1 * c(p65\_cyt) * c(I\kappa B\alpha\_nuc))$<br>$-I\kappa B\alpha\ import\ k_2 * c(I\kappa B\alpha\_nuc)$<br>$+I\kappa B\alpha\ import\ k_1 * c(I\kappa B\alpha\_cyt)$	
IκBα−p65_nuc	$-complex\ nculearshuttle\ k1 * c(I\kappa B\alpha - p65\_nuc)$<br>$-complex\ nculearshuttle\ k2 * c(I\kappa B\alpha - p65\_cyt)$<br>$V_{nucles} * (complex\ association\ k_1 * c(p65\_nuc) * c(I\kappa B\alpha\_nuc)$<br>$-complex\ association\ k_2 * c(I\kappa B\alpha - p65\_nuc)$<br>$-p65\_degradation\ k * c(I\kappa B\alpha - p65\_nuc)$<br>$-I\kappa B\alpha\ deg\ complex\ nuc\ k * c(I\kappa B\alpha - p65\_nuc)$	
IκBα-mRNA_I	$I\kappa B\alpha\ basal\ transcription\ k + I\kappa B\alpha\ transcription\ initiation\ k_A * c(p65\_nuc)$<br>-$I\kappa B\alpha\ basal\ transcription\ k * c(I\kappa B\alpha - mRNA\_I)$	
IκBα-mRNA_II	$I\kappa B\alpha\ basal\ transcription\ k * c(I\kappa B\alpha - mRNA\_I)$<br>-$I\kappa B\alpha\ basal\ transcription\ k * c(I\kappa B\alpha - mRNA\_II)$	
IκBα-mRNA_nuc	$I\kappa B\alpha\ basal\ transcription\ k * c(I\kappa B\alpha - mRNA\_II)$<br>$-I\kappa B\alpha - mRNA\ deg\ nuc\ k * c(I\kappa B\alpha mRNA\_nuc)$<br>$-I\kappa B\alpha - mRNA\ transport\ k * c(I\kappa B\alpha mRNA\ _nuc)$	
IκBα-mRNA_cyt	$-I\kappa B\alpha - mRNA\ deg\ cyt\ k * c(I\kappa B\alpha - mRNA\_cyt)$<br>$+I\kappa B\alpha - mRNA\ transport\ k * c(I\kappa B\alpha - mRNA\ _nuc)$	
p65-mRNA	–	fixed Value: 1 μM

Table 1: Ordinary differential equations describing all species in the original model. As initial concentrations the steady state concentrations were used.

Species	ODE	Initial Value
TNFα	-$V_{cytoplasm}$ * TNFa degradation k * c(TNFα)	intial value: 10 μM
IKKβ	$V_{cytoplasm} * (\frac{-IKK\beta\ active\ phsophorylation\ v_{max}*c(IKK\beta)*c(TNF\alpha)}{(IKK\beta\ active\ phosphorylation\ k_A*c(IKK\beta)+IKK\beta\ active\ phosphorylation\ k_M*c(IKK\beta)}$<br>$-IKK\beta\ basal\ phosphorylationk * c(IKK\beta)$<br>$+IKK\beta\ dephosphorylation\ k * c(IKK\beta\_p))$	modeled as a moiety<br>with pIKKβ<br>total concentration: 1 μM
IKKβ_p	1-IKKβ	
I$\kappa B\alpha$_cyt	$V_{cytoplasm} * (+I\kappa B\alpha\ translation\ k * c(I\kappa B\alpha\_mRNA\_cyt)$<br>$+p65\ degradation\ k * c(I\kappa B\alpha - p65\_cyt)$<br>$+complex\ association\ k_2 * c(complex\_cyt)$<br>$-I\kappa B\alpha\ deg\ cyt\ k * c(I\kappa B\alpha\_\mathrm{cyt})$<br>$-\frac{I\kappa B\alpha\ phosphorylation\ k_{cat}*c(IKK\beta)*c(I\kappa B\alpha\_cyt)}{I\kappa B\alpha\ phosphorylation\ k_M+c(I\kappa B\alpha\_cyt)}$<br>$-complex\ association\ k_1 * c(p65\_cyt) * c(I\kappa B\alpha\_cyt))$<br>$+I\kappa B\alpha\ import\ k2 * c(I\kappa B\alpha\_nuc)$<br>$-I\kappa B\alpha\ import\ k1 * c(I\kappa B\alpha\_cyt)$	
pSer536p65_cyt	$V_{cytoplasm} * (\frac{complexphosphorylation\ k_{cat}*c(IKK)*c(I\kappa B\alpha - p65\_cyt)}{complexphosphorylation\ k_M+c(I\kappa B\alpha - p65\_cyt)}$<br>$+p65\ translation\ k * c(p65mRNA)$<br>$+I\kappa B\alpha\ deg\ cyt\ k * c(I\kappa B\alpha - p65\_cyt)$<br>$-p65\ degradation\ k * c(p65\_cyt)$<br>$+complex\ association\ k_2 * c(I\kappa B\alpha - p65\_cyt)$<br>$-complex\ association\ k_1 * c(pSer536p65\_cyt) * c(I\kappa B\alpha\_cyt))$<br>$-p65\ nuclear\ shuttle\ k_1 * c(pSer536p65\_cyt)$<br>$+p65\ nuclear\ shuttle\ k_2 * c(pSer536p65\_nuc)$	
IκBα-p65_cyt	$+complex\ nuclear\ shuttle\ k_1 * c(I\kappa B\alpha - p65\_nuc)$<br>$-complex\ nuclear\ shuttle\ k_2 * c(I\kappa B\alpha - p65\_cyt)$<br>$V_{cytoplasm} * (complex\ association\ k_1 * c(pSer536p65\_cyt) * c(I\kappa B\alpha\_cyt)$<br>$-complex\ association\ k_2 * c(I\kappa B\alpha - p65\_cyt)$<br>$-\frac{complex\ phosphorylation\ k_{cat}*c(IKK)*c(I\kappa B\alpha - p65\_cyt)}{complex\ phosphorylation\ k_M+c(I\kappa B\alpha - p65\_cyt)}$<br>$-p65\ deg\ k * c(I\kappa B\alpha - p65\_cyt)$<br>$-I\kappa B\alpha\ deg\ complex\ cyt\ k * c(I\kappa B\alpha - p65\_cyt)$	
pIκBα	$V_{cytoplasm} * \frac{I\kappa B\alpha\ phosphorylation\ k_{cat}*c(IKK)*c(I\kappa B\alpha - p65\_cyt)}{I\kappa B\alpha\ phosphorylation\ k_M+c(I\kappa B\alpha - p65\_cyt)}$<br>$+\frac{complex\ phosphorylation\ k\_cat*c(IKK)*c(I\kappa B\alpha\_cyt)}{complex\ phosphorylation\ k_M+c(I\kappa B\alpha\_cyt)}$	

	$-I\kappa B\alpha\ p\ active\ degradation\ k * c(pI\kappa B\alpha)$	
pSer536p65_*nuc*	$V_{nucleus} * -p65\ degradation\ k * c(pSer536p65\_nuc)$<br>$-p65\ Ser536\ dephosphorylation\ k * c(pSer536p65\_nuc)$<br>$-\frac{pSer536p65\ MSK1\ phosphorylation\ k_{cat} * c(MSK1_active) * c(pSer536p65_nuc)}{pSer536p65\ MSK1\ phosphorylation\ k_M + c(pSer536p65_nuc)}$<br>$+p65\ shuttle\ to\ nucleus\ k1 * c(pSer536p65\_cyt)$<br>$-p65\ shuttle\ to\ nucleus\ k2 * c(pSer536p65\_nuc)$	
p65	$V_{nucleus} * I\kappa B\alpha\ deg\ complex\ nuc\ k * c(I\kappa B\alpha - p65\_nuc)$<br>$+p65\ Ser536\ dephosphorylation\ k * c(pSer536p65_nuc)$<br>$+p65\ Ser276\ dephosphorylation\ k * c(pSer276p65)$<br>$-p65\ degradation\ k * c(p65)$<br>$-\frac{p65\ MSK1\ phosphorylation\ k_{cat} * c(MSK1_active) * c(p65)}{p65\ MSK1\ phosphorylation\ k_M + c(p65)}$<br>$+complex\ association\ k_2 * c(I\kappa B\alpha - p65\_nuc)$<br>$-complex\ association\ k_1 * c(p65\_nuc) * c(I\kappa B\alpha\_nuc))$	
pSer276p65_*nuc*	$V_{nucleus} * -p65\ degradation\ k * c(pSer276p65\_nuc)$<br>$-p65\ Ser276\ dephosphorylation\ k * c(pSer276p65_nuc)$<br>$+\frac{pSer536p65\ MSK1\ phosphorylation\ k_{cat} * c(MSK1_active) * c(pSer536p65_nuc)}{pSer536p65\ MSK1\ phosphorylation\ k_M + c(pSer536p65_nuc)}$<br>$+\frac{p65\ MSK1\ phosphorylation\ k_{cat} * c(MSK1_active) * c(p65)}{p65\ MSK1\ phosphorylation\ k_M + c(p65)}$	
IκBα_*nuc*	$V_{nucleus} * +p65\ degradation\ k * c(I\kappa B\alpha - p65\_nuc)$<br>$+complex\ association\ k_2 * c(I\kappa B\alpha - p65)$<br>$-I\kappa B\alpha\ deg\ cyt\ k * c(I\kappa B\alpha\_\mathrm{cyt})$<br>$-complex\ association\ k_1 * c(p65\_cyt) * c(I\kappa B\alpha\_nuc))$<br>$-I\kappa B\alpha\ import\ k_2 * c(I\kappa B\alpha\_nuc)$<br>$+I\kappa B\alpha\ import\ k_1 * c(I\kappa B\alpha\_cyt)$	
IκB$\alpha-$p65_nuc	$-complex\ nculearshuttle\ k1 * c(I\kappa B\alpha - p65\_nuc)$<br>$-complex\ nculearshuttle\ k2 * c(I\kappa B\alpha - p65\_cyt)$<br>$V_{nucles} * (complex\ association\ k_1 * c(p65) * c(I\kappa B\alpha\_nuc)$<br>$-complex\ association\ k_2 * c(I\kappa B\alpha - p65\_nuc)$<br>$-p65\_degradation\ k * c(I\kappa B\alpha - p65\_nuc)$<br>$-I\kappa B\alpha\ deg\ complex\ nuc\ k * c(I\kappa B\alpha - p65\_nuc)$	
IκBα-mRNA_I	$I\kappa B\alpha\ basal\ transcription\ k + I\kappa B\alpha$<br>$transcription\ initiation\ k_{A1} * c(pSer536p65\_nuc)$<br>$+I\kappa B\alpha\ transcription\ initiation\ k_{A2} * c(p65)$<br>$+I\kappa B\alpha\ transcription\ initiation\ k_{A3} * c(pSer276p65)$	

	-$I\kappa B\alpha\ basal\ transcription\ k * c(I\kappa B\alpha - mRNA\_I)$	
IκBα-mRNA_II	$I\kappa B\alpha\ basal\ transcription\ k * c(I\kappa B\alpha - mRNA\_I)$<br>-$I\kappa B\alpha\ basal\ transcription\ k * c(I\kappa B\alpha - mRNA\_II)$	
IκBα-mRNA_nuc	$I\kappa B\alpha\ basal\ transcription\ k * c(I\kappa B\alpha - mRNA\_II)$<br>$-I\kappa B\alpha - mRNA\ deg\ nuc\ k * c(I\kappa B\alpha mRNA\_nuc)$<br>$-I\kappa B\alpha - mRNA\ transport\ k * c(I\kappa B\alpha mRNA\ _nuc)$	
IκBα-mRNA_cyt	$-I\kappa B\alpha - mRNA\ deg\ cyt\ k * c(I\kappa B\alpha - mRNA\_cyt)$<br>$+I\kappa B\alpha - mRNA\ transport\ k * c(I\kappa B\alpha - mRNA\ _nuc)$	
p65-mRNA	–	fixed Value: 1 μM
nosignal	1 - signal1 - signal2	
signal1	$V_{cytoplasm} * (\frac{(MSK1\ signal\ transmission\ v\_max * c(nosignal) * c(TNF\alpha)}{(MSK1\ signal\ transmission\ k_M + c(nosignal)) * (MSK1\ signal\ transmission\ k_A * c(TNF\alpha)}$<br>$MSK1\ signal\ transmission\ 2 * c(signal1))$	
signal2	$V_{cytoplasm} * (-MSK1\ signal\ transmission\ 2 * c(signal1)$<br>$MSK1\ signal\ depletion * c(signal2))$	
MSK1_active	$V_{cytoplasm} * (\frac{MSK1\ phosphoryaltion\ v_{max} * c(MSK1\_unactive)}{MSK1\ phosphoryaltion\ k_M + c(MSK1\_unactive)}$<br>$MSK1\ deactivationk * c(MSK1\_active))$	
MSK1_unactive	1 - MSK1_active	

Table 2: Ordinary differential equations describing all species in the model expanded by p65 phosphorylaiton. As initial concentrations the steady state concentrations were used.

Parameter	Number	Lower Fitting Bound	Upper Fitting Bound	Reference
$V_{cytoplasm}$	–	fixed Value:	4	
$V_{nucleus}$	–	fixed Value:	1	
complex association k_1	1	5.90E+003	590184	[1, 2]
IκBα complex association k_2	2	7.26E-005	0.0072554	[1, 2]
IκBα complex phosphorylation k_{cat}	3	1.22E-001	1.22E+001	
complex phosphorylation k_M	4	1.70E-006	1.70E-004	
complex shuttle k_1	5	0.000181258	0.0181258	[1, 2]
complex shuttle k_2	6	0.0626749	6.26749	[1, 2]
IκBα degradation cyt k_1	7	48.0121	4801.21	measured: $1.82 * 10^{-5}$, [1, 2]
IκBα degradation complex c k_1	8	1.78E-007	3.00E-005	measured: $1.82 * 10^{-5}$,[1, 2]
IκBα degradation nuc complex k_1	9	1.70E-007	3.00E-005	measured: $1.82 * 10^{-5}$, [1, 2]
IκBα degradation nuc k	10	1.11E-007	0.005	measured: $1.82 * 10^{-5}$, [1, 2]
IκBα mRNA degradation cyt k	11	1.30E-006	0.005	[1, 2]
IκBα mRNA degradation nuc k	12	6.78E-005	0.00678313	[1, 2]
IκBα mRNA transport k	13	1.09E-005	0.00109224	–
IκBα p degradation k	14	0.00668578	0.668578	[1]
IκBα phosphorylation k_{cat}	15	1.00E-003	129.33	–
IκBα phosphorylation k_M	16	1.00E-003	1209.81	–
IκBα nuclear shuttle k_1	17	0.000560725	0.0560725	[1, 2]
IκBα nuclear shuttle k_2	18	8.88524	888.524	[1, 2]
IκBα basal transcription k	19	0.361549	36.1549	–
IκBα transcription k_A	20	0.123087	12.3087	–
IκBα translation k	21	0.0624634	6.24634	[1, 2]
IKKβ active phosphorylation k_A	22	0.214977	21.4977	–
IKKβ active phosphorylation k_M	23	0.000243324	0.0243324	–
IKKβ active phosphorylation v_{max}	24	2.10E-007	0.9	–
IKKβ basal phosphorylation k	25	4543.75	454375	–
IKKβ dephosphorylation k	26	0.912083	91.2083	
k IκBα-mRNA (scaling factor, scaling factor, experimental set1):	27	0.0279505	2.79505	–
k IκBα-mRNA (scaling factor, experimental set 2):	28	0.00260405	0.260405	–
k pIκBα (scaling factor, experimental set 1):	29	0.000891645	0.0891645	–

k pIκBα (scaling factor, experimental set 2):	30	3.8571	385.71	–
k pIκBα (scaling factor, experimental set 3):	31	0.0935347	9.35347	–
k pIκBα (scaling factor, experimental set4):	32	2.26E-005	0.00226192	–
k IκBα (scaling factor, experimental set 1):	33	3.06E-005	0.003062	–
k IκBα (scaling factor, experimental set 2):	34	0.0756102	7.56102	–
k IκBα (scaling factor, experimental set 3):	35	1.00E-006	0.01165	–
k IκBα (scaling factor, experimental set 4):	36	0.000135806	0.0135806	–
k pp65 (scaling factor, experimental set 1):	37	5.85E-007	5.85E-005	–
k pp65 (scaling factor, experimental set 2):	38	0.000251125	0.0251125	–
k pp65 (scaling factor, experimental set 3):	39	0.8	80	–
k pp65 (scaling factor, experimental set 4):	40	5.00E-007	5.00E-005	–
p65 degradation k_1	41	0.00654147	0.654147	measured: $4.77 * 10^{-6}$
p65 shuttle k_1	42	8.10E-006	0.000809971	[1, 2]
p65 shuttle k_2	43	1.73E-007	1.73E-005	[1, 2]
p65 translation k	44	0.00807252	0.807252	
TNFα degradation k_1	45	119.238	11923.8	

Table 3: Parameters and fitting boundaries of the parameters of the original model

Parameter	Number	Lower Fitting Bound	Upper Fitting Bound	Reference
$V_{cytoplasm}$	–	fixed Value:	4	
$V_{nucleus}$	–	fixed Value:	1	
complex association k_1	1	7.56102	0.0756102	[1, 2]
complex association k_2	2	0.01165	1.00E-006	[1, 2]
complex nuclear shuttle k_1	3	0.0135806	0.000135806	[1, 2]
complex nuclear shuttle k_2	4	5.85E-005	5.85E-007	[1, 2]
phosphorylation k_{cat}	5	4801.21	48.0121	–
complex phosphorylation k_M	6	6.26749	0.0626749	–
IκBα deg complex cyt k	7	3.00E-005	1.78E-007	[1, 2]
IκBα deg complex nuc k	8	3.00E-005	1.70E-007	[1, 2]
IκBα deg cyt k	9	0.005	1.11E-007	[1, 2]
IκBα deg nuc k	10	0.005	1.30E-006	[1, 2]
IκBα mRNA deg cyt k	11	0.00678313	6.78E-005	[1, 2]
IκBα mRNA deg nuc k	12	0.00109224	1.09E-005	[1, 2]
IκBα mRNA transport k	13	0.668578	0.00668578	–
IκBα nuclear shuttle k_1	14	129.33	1.00E-003	[1, 2]
IκBα nuclear shuttle k_2	15	1209.81	1.00E-003	[1, 2]
IκBα p active degradation k	16	0.0560725	0.000560725	[1]
IκBα phosphorylation $k_c at$	17	36.1549	0.361549	–
IκBα phosphorylation k_M	18	888.524	8.88524	–
IκBα transcription k_A1 pp65	19	21.4977	0.214977	–
IκBα transcription k_A2 p65	20	12.3087	0.123087	–
IκBα transcription k_A3 pp65Ser237	21	6.24634	0.0624634	–
IκBα basal transcription k	22	0.0243324	0.000243324	–
IκBα translation k	23	0.9	2.10E-007	[1, 2]
IKKβ active phosphorylation k_A	24	590184	5.90E+003	–
IKKβ active phosphorylation k_M	25	0.0072554	7.26E-005	–
IKKβ active phosphorylation v_{max}	26	1.22E+001	1.22E-001	–
IKKβ basal phosphoylation k	27	1.70E-004	1.70E-006	–
IKKβ dephopshorylation k	28	0.0181258	0.000181258	–
k IκBα mRNA (scaling factor, experiment data set 1)	29	0.0559949	0.000559949	–
k IκBα mRNA (scaling factor, experiment data set 2)	30	0.0559949	0.000559949	–
k IκBα p (scaling factor, experimental data set 1)	31	1990.79	19.9079	–

k IκBα p (scaling factor, experimental data set 2)	32	1990.79	19.9079	–
k IκBα p (scaling factor, experimental data set 3)	33	1990.79	19.9079	–
k IκBα p (scaling factor, experimental data set 4)	34	1990.79	19.9079	–
k IκBα (scaling factor, experimental data set 1)	35	412.126	4.12126	–
k IκBα (scaling factor, experimental data set 2)	36	412.126	4.12126	–
k IκBα (scaling factor, experimental data set 3)	37	412.126	4.12126	–
k IκBα (scaling factor, experimental data set 4)	38	412.126	4.12126	–
k pp65 (scaling factor, experiemtnal data set 1)	39	3428.35	34.2835	–
k pp65 (scaling factor, experiemtnal data set 2)	40	3428.35	34.2835	–
k pp65 (scaling factor, experiemtnal data set 3)	41	3428.35	34.2835	–
k pp65 (scaling factor, experiemtnal data set 4)	42	3428.35	34.2835	–
MSK1 activation k_{cat}	43	91.2083	0.912083	–
MSK1 activation k_M	44	454375	4543.75	–
MSK1 deactivation	45	2.79505	0.0279505	–
MSK1 signal depletion k	46	0.260405	0.00260405	–
MSK1 signal transmission 2	47	0.0891645	0.000891645	–
MSK1 signal transmission k_A	48	385.71	3.8571	–
MSK1 signal transmission k_M	49	9.35347	0.0935347	–
MSK1 signal transmission v_{max}	50	0.00226192	2.26E-005	–
p65 degradation k	51	5.00E-005	5.00E-007	measured: $4.76512 * 10^{-6}$
p65 MSK1 phosphorylation k_M	52	0.0251125	0.000251125	–
p65 MSK1 phosphrylation k_{cat}	53	80	0.8	–
p65 nuclear shuttle k_1	54	0.654147	0.00654147	[1, 2]
p65 nuclear shuttle k_2	55	0.000809971	8.10E-006	[1, 2]
p65 translation k	56	1.73E-005	1.73E-007	
pSer276p65 dephosphorylation	57	0.0379123	0.000379123	–
pSer276p65 MSK1 phosphrylation k_{cat}	58	11923.8	119.238	–
pSer276p65 MSK1 phosphrylation k_M	59	0.807252	0.00807252	–
pp65 Ser237 dephosphorylation	60	0.120938	0.00120938	–
TNFα degradation k	61	0.003062	3.06E-005	–

Table 4: Parameters and fitting boundaries of the parameters of the expanded model

Parameter \ Fit	1	2	3	4	5	6	7	8	9	10
Obj. Fun. Value	19591.3	21077.3	21215.5	21330.5	21410.6	21426.7	21532	21588.3	21588.5	21635.9
1	8500.63	8497.79	3504.14	259.224	4426.5	8515.22	1387.47	394.494	3120.04	2857.69
2	7.79623	31.4439	32.2863	7.27949	56.6869	53.7332	30.1105	4.39496	23.7959	38.4073
3	51.7339	0.534	10.2855	2.13462	0.517339	21.3937	51.7302	0.521416	50.0001	0.521846
4	3444.65	3432.68	3420	3355.58	3369.27	3429.22	72.1914	49.6176	1475	376.305
5	1.05256	2.42811	0.0768908	0.194523	0.0628975	2.04844	0.23346	0.207735	2.42013	0.216382
6	0.0191731	0.000208822	0.000363915	0.00125637	0.00371114	0.0151125	2.33852e-05	0.00129274	0.00100228	0.000416757
7	2.82089e-06	1.93479e-07	2.90969e-07	2.42946e-07	2.50263e-07	1.13432e-06	5.12432e-05	0.00122947	0.0690014	3.75251e-06
8	1.83757e-06	1.81822e-06	8.75257e-06	6.54726e-06	1.93989e-05	6.12019e-06	1.83939e-06	7.93376e-06	0.00158715	2.07566e-06
9	1.86192e-06	1.81792e-06	1.91142e-06	1.8177e-06	1.81877e-06	4.67875e-06	1.90003e-06	2.36879e-06	0.0015137	1.95548e-06
10	4.79046e-06	5.59777e-06	0.00019904	4.79356e-06	0.000214809	0.000333662	8.13721e-05	4.85177e-06	3.54477e-05	0.000356544
11	0.0198431	0.00810722	0.0371886	0.0166845	0.0265983	0.0371965	0.0325858	0.037169	0.0096474	0.0196141
12	1.80444e-06	1.7301e-06	0.000157599	1.76401e-06	0.000167612	1.74859e-06	4.55433e-06	0.000112992	4.86459e-06	1.73216e-06
13	0.000215566	0.000196502	0.00984995	9.86413e-05	0.00949634	9.86075e-05	0.00964623	0.00983372	0.000132919	9.86055e-05
14	0.00288599	0.00277388	0.00247444	0.00218317	0.00248771	0.00310691	0.00200348	0.00273859	0.0233277	0.00282463
15	35.6693	51.732	10.4842	51.7272	51.7056	32.9318	11.9745	6.53376	50.3851	17.9328
16	180.762	647.072	82.1244	144.873	108.933	416.331	72.4508	405.547	1001.81	80.0393
17	18.995	14.2058	5.6965	7.99889	26.7312	21.0733	35.9289	1.6322	0.999683	13.9487
18	0.0764076	0.242995	0.0177568	0.0382271	0.0236488	0.390654	0.0804749	0.0484203	0.0437894	0.0804084
19	0.00777451	0.00777115	0.000228102	0.00776794	0.000225364	0.00120719	0.000270755	0.000214904	0.000230962	0.00101948
20	1.01725	0.712306	1.69567	8.42409	0.712099	0.71209	5.24588	2.02299	1.44461	5.76752
21	0.000759844	0.00023208	0.00157973	0.000235581	0.00206129	0.00598087	0.000361327	0.00255629	0.0194676	0.000339934
22	14204.2	214236	2146.5	92006.1	2142.45	6476.78	2189.89	2148.28	2900.33	128584
23	0.000110369	0.000110758	0.000110776	0.000187214	0.000110543	0.000110364	0.000110408	0.000110433	0.000110386	0.000122403
24	5.53661	75.4	75.3989	38.1022	75.3992	1.7997	75.2613	75.3627	21.1184	49.0737
25	0.000460081	0.000286666	0.00268993	0.000360673	0.00287019	0.000319364	0.00287027	0.00215059	0.00136386	0.000384486
26	0.00248347	0.00216094	0.00888233	0.00385796	0.00968767	0.00340333	0.0112622	0.00655836	0.00319709	0.00331221
27	0.61259	0.010012	0.629042	0.636138	0.0126894	0.00925664	0.0065819	0.636301	0.632984	0.634916
28	0.636072	0.569874	0.632344	0.635982	0.635661	0.0173664	0.63637	0.609747	0.623714	0.636358
29	344.33	435.152	381.074	148.162	218.201	336.805	280.74	286.823	536.587	192.412
30	275.889	343.694	300.996	114.178	171.349	272.018	221.877	228.784	416.893	149.169
31	458.298	575.321	500.553	197.32	284.29	444.702	367.596	375.541	716.325	249.203
32	304.455	377.526	332.674	129.272	190.421	298.995	249.216	252.383	476.779	166.304
33	158.079	189.985	136.591	67.8597	70.7436	116.019	125.008	95.2454	215.126	67.5873
34	159.921	193.136	135.236	68.0836	70.2617	115.884	124.071	94.3593	212.233	66.8513
35	159.486	193.006	139.216	69.6139	72.2413	116.332	129.3	95.962	215.988	69.2738
36	152.222	184.638	132.422	65.7809	68.4789	111.161	120.618	92.2915	206.512	65.267
37	3375.25	2066.48	2854.53	2356.55	1075.59	2677.3	2905.58	3661.2	1854.12	1163.75
38	2655.32	1604.85	2278.7	1862.86	850.991	2123.79	2318.14	2898.03	1458.41	911.758
39	2556.17	1535.83	2165.65	1731.4	813.993	2016.73	2208.76	2803.8	1382.2	868.313
40	2942.92	1803.91	2492.48	2046.73	937.684	2336.98	2550.8	3192.39	1662.12	1009.05
41	1.5762e-05	2.59338e-05	1.03587e-05	2.71803e-05	5.13038e-06	2.5022e-05	1.04056e-05	7.60988e-06	1.46776e-05	9.02364e-06

42	7.0848	11.4178	3.40886	4.28408	2.74593	53.9497	8.44063	11.1844	3.75065	7.83606
43	0.000129752	0.000128055	9.59586e-05	0.00146341	8.26395e-05	0.0759265	0.067079	0.0797552	8.1523e-05	0.078073
44	2.20984e-07	4.6243e-07	2.91785e-07	5.80118e-07	3.93634e-07	7.18677e-07	2.03821e-07	2.14535e-07	2.87163e-07	5.90237e-07
45	0.0028817	0.00408391	0.00747277	0.00234342	0.00730356	0.00387089	0.006773	0.00836174	0.00529174	0.00379588

Table 5: Objective function value and exact values of all parameters fitted via particle swarm in the parameter estimation starting from random start values. Parameter Sets 1 - 10 for the original model. The parameters are: complex association k_1 – 1, IκBα complex association $k4_2$ – 2, IκBα complex phosphorylation k_{cat} – 3, complex phosphorylation k_M – 4, complex shuttle k_1 – 5, complex shuttle k_2 – 6, IκBα degradation cyt k_1 – 7, IκBα degradation complex c k_1 – 8, IκBα degradation nuc complex k_1 – 9, IκBα degradation nuc k – 10, IκBα mRNA degradation cyt k – 11, IκBα mRNA degradation nuc k – 12, IκBα mRNA transport k – 13, IκBα p degradation k – 14, IκBα phosphorylation k_{cat} – 15, IκBα phosphorylation k_M – 16, IκBα nuclear shuttle k_1 – 17, IκBα nuclear shuttle k_2 – 18, IκBα basal transcription k – 19, IκBα transcription k_A – 20, IκBα translation k – 21, IKKβ active phosphorylation k_A – 22, IKKβ active phosphorylation k_M – 23, IKKβ active phosphorylation v_{max} – 24, IKKβ basal phosphorylation k – 25, IKKβ dephosphorylation k – 26, k IκBα-mRNA (scaling factor, scaling factor, experimental set1): – 27, k IκBα-mRNA (scaling factor, experimental set 2): – 28, k pIκBα (scaling factor, experimental set 1): – 29, k pIκBα (scaling factor, experimental set 2): – 30, k pIκBα (scaling factor, experimental set 3): – 31, k pIκBα (scaling factor, experimental set4): – 32, k IκBα (scaling factor, experimental set 1): – 33, k IκBα (scaling factor, experimental set 2): – 34, k IκBα (scaling factor, experimental set 3): – 35, k IκBα (scaling factor, experimental set 4): – 36, k pp65 (scaling factor, experimental set 1): – 37, k pp65 (scaling factor, experimental set 2): – 38, k pp65 (scaling factor, experimental set 3): – 39, k pp65 (scaling factor, experimental set 4): – 40, p65 degradation k1 – 41, p65 shuttle k1 – 42, p65 shuttle k2 – 43, p65 translation k – 44, TNFα degradation k1 – 45.

Parameter \ Fit	11	12	13	14	15	16	17	18	19	20
Obj. Fun. Value	21652.5	21727	21727	21880.4	21948.9	22065.8	22428	22429.5	22539.6	22569.4
1	2083.28	4933.06	4181.44	730.382	1167.3	5352.14	637.896	210.312	2119.04	6652.59
2	66.0199	106.36	103.612	13.0055	9.90118	26.8052	34.9062	2.18927	43.5584	85.1342
3	0.557672	51.6465	0.517339	0.718734	51.7195	41.2737	0.67298	0.518351	51.7154	2.34875
4	1094.29	2420.66	2090.86	3202.33	3170.74	573.539	3133.73	3455	791.974	1414.36
5	0.784399	0.815173	0.715771	0.0676	0.49313	0.380675	0.906871	0.19727	1.48126	0.966106
6	6.72201e-05	0.0199849	0.00840767	0.000178268	0.00482085	3.56766e-05	0.02	0.0194872	0.0169645	0.0015723
7	2.42988e-06	4.58823e-07	6.64379e-06	1.65537e-07	1.15211e-05	6.28732e-06	1.75945e-07	2.16221e-07	6.92128e-06	2.11613e-06
8	1.81782e-06	2.0157e-06	2.37296e-06	1.82053e-05	4.17895e-06	1.96324e-06	0.000223177	2.09642e-06	3.72195e-05	1.81785e-06
9	2.02608e-06	0.000167692	1.82167e-06	1.83113e-06	4.00405e-06	1.86314e-06	2.13e-06	1.82521e-06	2.03577e-05	2.50164e-06
10	0.000386363	4.79016e-06	0.000449863	0.000646767	0.000595034	0.000513008	4.80558e-06	0.000519692	1.20847e-05	0.000761466
11	0.0021282	0.0254455	0.00608325	0.0371967	0.0250717	0.037177	0.0125054	0.0371961	0.034539	0.0371939
12	1.91334e-06	4.80489e-05	2.47112e-05	1.73453e-06	1.74897e-06	0.000164596	1.82273e-06	2.2131e-06	7.78196e-05	0.000125559
13	0.00985959	0.00977791	0.00705555	9.86089e-05	9.86045e-05	0.00237552	0.000150812	0.00981825	9.87731e-05	0.00201446
14	0.00270193	0.00267377	0.00302332	0.00387736	0.00360501	0.00327762	0.00240212	0.00339089	0.00344975	0.00369852
15	11.6987	5.66488	12.2705	39.8912	6.08831	51.7133	30.4034	46.038	25.2293	26.9486
16	111.917	834.527	246.888	365.863	174.442	550.908	295.191	359.521	1850.03	413.019
17	20.1292	0.743468	10.7188	2.41769	6.23229	12.4237	11.8351	2.32178	24.6915	2.72991
18	0.163379	17.982	0.130894	0.0217992	0.120018	0.108032	0.171587	0.0253498	0.150593	0.127873
19	0.000217195	0.000413469	0.000191603	0.000695796	0.000771515	0.000192553	0.00776238	7.77457e-05	0.00777457	0.000170484
20	12.1645	2.83976	7.77283	12.0968	3.9779	2.96821	4.97825	2.11027	0.715493	5.6979
21	2.45163e-05	0.00314758	0.000124406	0.000736598	0.00117042	0.00185273	0.000111314	0.00348459	0.00127653	0.0016927
22	10824.2	44796.2	26264.8	19563.8	111017	152028	14201.2	40371.4	2142.36	55479.3
23	0.000110367	0.000408527	0.00011357	0.000115659	0.000111273	0.0109462	0.000110484	0.00011911	0.000113804	0.000200222
24	5.99183	15.5847	16.9269	7.07454	50.633	50.2362	5.74703	9.0985	75.3282	6.75273
25	0.000569626	0.000353705	0.000772853	0.000478224	0.000579551	0.00042432	0.000429724	0.000289373	0.00269278	0.000166012
26	0.00296411	0.00297057	0.00293768	0.00315051	0.00285855	0.00336758	0.00372774	0.00309445	0.00640063	0.00348914
27	0.058134	0.633796	0.272599	0.636052	0.636311	0.00643004	0.635922	0.635328	0.0063673	0.632615
28	0.0421709	0.00636859	0.236954	0.478647	0.604961	0.0113773	0.63637	0.635519	0.00637245	0.628219
29	111.505	157.088	127.37	82.9676	265.714	453.799	213.027	253.894	419.884	230.385
30	87.1216	123.672	99.2526	64.9634	205.02	353.142	162.151	199.812	343.276	182.171
31	146.879	208.593	166.204	108.62	346.891	600.329	287.658	340.967	552.102	303.937
32	97.9447	140.471	111.085	72.8517	232.492	395.762	187.915	225.122	379.405	201.463
33	45.0733	68.981	46.4664	23.2743	80.581	146.301	101.044	97.0847	118.96	65.211
34	44.5922	69.9431	46.221	22.7856	80.5411	145.684	101.519	102.02	123.159	65.3358
35	46.1037	69.1607	47.5522	23.7094	82.3628	148.871	102.104	98.3503	123.298	66.3973
36	43.6864	65.1852	44.8399	22.2301	79.2126	140.859	97.66	94.698	115.466	63.278
37	1280.94	1969.94	1300.33	690.182	2046.77	3276.34	1892.66	2092.55	1765.66	1903.3
38	1015.71	1555.29	1030.35	533.283	1640.62	2596.43	1499.49	1645.52	1394.7	1527.69

39	967.472	1474.98	983.201	519.613	1555.41	2445.94	1378.19	1572.17	1324	1448.03
40	1110.89	1732.43	1132.17	602.63	1790.54	2858.88	1610	1812.26	1492.69	1661.47
41	3.31179e-06	7.03016e-06	6.4512e-06	1.05594e-06	6.94268e-06	3.53436e-05	1.02935e-05	7.49782e-06	1.71214e-05	7.03041e-06
42	30.538	0.0221753	27.9999	4.46758	19.3193	17.4998	16.8968	5.60445	83.03	22.8567
43	0.000436374	0.0797583	0.000220736	0.000444138	9.64204e-05	0.0787	0.08	0.00135749	0.000101496	0.000230617
44	1.9061e-07	2.09696e-07	3.99736e-07	2.1674e-07	3.34787e-07	1.04149e-06	1.8968e-07	2.5927e-07	6.88591e-07	3.13451e-07
45	0.00366564	0.00337753	0.00359902	0.00345414	0.00364386	0.00346145	0.00143103	0.00373793	0.00868691	0.00371248

Table 6: Objective function value and exact values of all parameters fitted via particle swarm in the parameter estimation starting from random start values. Parameter Sets 11 - 20 for the original model. The parameters are: complex association k_1 – 1, IκBα complex association $k4_2$ – 2, IκBα complex phosphorylation k_{cat} – 3, complex phosphorylation k_M – 4, complex shuttle k_1 – 5, complex shuttle k_2 – 6, IκBα degradation cyt k_1 – 7, IκBα degradation complex c k_1 – 8, IκBα degradation nuc complex k_1 – 9, IκBα degradation nuc k – 10, IκBα mRNA degradation cyt k – 11, IκBα mRNA degradation nuc k – 12, IκBα mRNA transport k – 13, IκBα p degradation k – 14, IκBα phosphorylation k_{cat} – 15, IκBα phosphorylation k_M – 16, IκBα nuclear shuttle k_1 – 17, IκBα nuclear shuttle k_2 – 18, IκBα basal transcription k – 19, IκBα transcription k_A – 20, IκBα translation k – 21, IKKβ active phosphorylation k_A – 22, IKKβ active phosphorylation k_M – 23, IKKβ active phosphorylation v_{max} – 24, IKKβ basal phosphorylation k – 25, IKKβ dephosphorylation k – 26, k IκBα-mRNA (scaling factor, scaling factor, experimental set1): – 27, k IκBα-mRNA (scaling factor, experimental set 2): – 28, k pIκBα (scaling factor, experimental set 1): – 29, k pIκBα (scaling factor, experimental set 2): – 30, k pIκBα (scaling factor, experimental set 3): – 31, k pIκBα (scaling factor, experimental set4): – 32, k IκBα (scaling factor, experimental set 1): – 33, k IκBα (scaling factor, experimental set 2): – 34, k IκBα (scaling factor, experimental set 3): – 35, k IκBα (scaling factor, experimental set 4): – 36, k pp65 (scaling factor, experimental set 1): – 37, k pp65 (scaling factor, experimental set 2): – 38, k pp65 (scaling factor, experimental set 3): – 39, k pp65 (scaling factor, experimental set 4): – 40, p65 degradation k1 – 41, p65 shuttle k1 – 42, p65 shuttle k2 – 43, p65 translation k – 44, TNFα degradation k1 – 45.

Parameter \ Fit	21	22	23	24	25	26	27	28	29	30
Obj. Fun. Value	22671.1	22679.3	22693.1	22795	22904	23005	23109.9	23169.1	23176.8	23213.3
1	8515.11	3083.05	2361.29	543.252	2206.58	8462.2	5051.4	7140.03	212.072	3520.08
2	110.621	19.0802	58.3218	165.837	29.3027	150.962	464.922	43.9595	26.707	283.741
3	51.7168	0.517699	47.3766	0.51769	3.09951	40.9652	0.522221	51.7139	14.6746	0.519796
4	548.867	3250.78	712.154	314.597	98.7846	371.41	2029.13	523.179	34.6293	2518.85
5	0.550514	0.174399	0.181015	1.26646	0.190454	2.41597	2.43141	2.43326	0.288756	1.0513
6	0.0199852	0.000159987	0.00933405	2.14003e-05	0.00631144	0.0199621	0.00375984	0.0199059	0.00185335	0.0049727
7	1.60288e-06	1.45625e-05	2.82079e-07	1.66814e-06	3.36498e-07	1.64953e-06	1.7212e-07	0.00050323	1.89072e-07	5.68854e-07
8	0.00181092	1.83135e-06	7.32028e-06	1.73466e-05	1.98833e-06	0.000117473	2.36693e-06	0.000709086	1.819e-06	1.91933e-06
9	4.12696e-06	3.33528e-06	0.000128248	1.81769e-06	0.00156661	1.81953e-06	1.82007e-06	0.00162161	1.88352e-06	1.81844e-06
10	0.000482567	0.000898048	5.04299e-06	4.79247e-06	5.20091e-06	0.0004973	4.79678e-06	4.80721e-06	4.79298e-06	4.79017e-06
11	0.0222491	0.0368773	0.012496	0.00554212	0.02706	0.0128612	0.0170252	0.0140474	0.0090707	0.0277379
12	0.000172996	0.000172888	0.000138488	0.000172433	0.000150132	0.000172939	1.68371e-05	0.000168599	0.000172919	0.000173006
13	0.000100301	0.000990229	0.00100562	0.00122727	0.000918035	0.000873602	0.00170231	0.00118631	0.00502737	0.00180282
14	0.00333774	0.00434946	0.00297742	0.00235795	0.00294766	0.00309861	0.00245663	0.0035789	0.0024957	0.00244536
15	0.517361	35.3596	4.92292	51.7304	3.52461	9.20787	22.6532	5.73164	51.6967	51.7339
16	113.713	488.609	3455.32	191.592	1863.91	1884.16	159.019	684.945	73.0038	350.243
17	0.145111	3.92616	9.09546	33.3928	6.64792	3.81392	35.9612	35.9578	35.9271	35.9394
18	17.9859	0.0423569	8.17692	0.163053	2.9099	4.26267	0.496372	4.96399	0.109744	0.27969
19	0.00165573	0.000183076	0.000930436	0.00098814	0.000864538	0.000668292	0.000888103	0.000514953	0.000694495	0.000836752
20	3.09858	14.4606	2.24921	1.31687	9.41634	2.00818	3.66672	34.1208	3.01257	3.46234
21	0.00287757	0.000498583	0.00786174	0.000247009	0.0116093	0.0119378	0.000105022	0.0180734	6.46206e-05	0.000144891
22	41755.4	107063	37537.9	26260.3	42622.4	91635.5	35825.3	27501.6	11407.9	37586
23	0.000111126	0.000110723	0.000110916	0.000111966	0.000139881	0.000110979	0.0109231	0.000110589	0.000110399	0.000110405
24	5.70791	35.8164	15.3303	12.3194	22.7327	15.2075	10.0482	4.21349	3.06241	17.4247
25	0.00018197	0.000510957	0.000596794	0.000425737	0.000756954	0.000221039	0.000270157	0.000263207	0.000255643	0.000449362
26	0.00276899	0.00298505	0.00285941	0.00428489	0.00278667	0.00306489	0.00408648	0.00350599	0.00396011	0.0039413
27	0.586431	0.633533	0.633883	0.530269	0.015216	0.00636547	0.634591	0.57808	0.332324	0.63637
28	0.0122755	0.635935	0.635511	0.457544	0.63595	0.0568826	0.636052	0.417592	0.264042	0.636151
29	246.621	313.026	169.859	70.4884	242.688	224.795	255.546	240.953	169.952	286.753
30	193.352	245.821	133.53	52.1457	190.24	177.172	192.278	190.669	126.966	215.55
31	335.078	418.385	226.346	91.8709	325.565	303.436	336.649	323.727	222.713	379.423
32	219.272	279.21	151.607	61.8056	215.458	200.72	225.291	215.272	149.102	252.871
33	111.962	81.4433	82.2907	35.4068	113.973	102.939	123.178	103.469	79.07	137.175
34	114.336	81.4048	83.2866	35.7587	115.141	103.995	125.166	104.658	79.7052	138.395
35	111.852	83.2592	82.8471	35.4703	115.29	104.217	124.004	103.86	79.8537	137.942
36	106.871	79.4708	78.6415	33.4685	109.338	98.1236	117.853	98.568	75.5827	131.137
37	2058.98	1401.34	2456.5	914.922	2314.36	2505.64	1489.13	2482.09	813.919	1211.19
38	1637.56	1098.32	1916.78	683.236	1817.44	1970.92	1114.74	1937.85	611.478	901.212

39	1528.53	1051.14	1822.97	654.461	1718	1872.02	1057	1857.1	579.726	857.098
40	1776.99	1204.81	2116.47	772.362	2004.74	2174.34	1248.88	2146	687.725	1020.85
41	1.49676e-05	2.3568e-05	1.10287e-05	2.59476e-05	2.60577e-05	1.27144e-05	1.98307e-05	1.13321e-05	3.021e-06	2.04943e-05
42	0.0285295	6.50298	0.000833541	23.4145	0.000830459	0.0527455	48.1944	0.00520256	5.88238	19.5549
43	0.0797723	0.000184217	8.01863e-05	0.000427248	0.0799982	0.0773037	0.0338794	0.0485689	0.0799771	0.00127486
44	3.51131e-07	1.62665e-06	1.89088e-07	8.19468e-07	4.89963e-07	2.34945e-07	3.73608e-07	2.52659e-07	1.09498e-07	4.71682e-07
45	0.00379263	0.00344742	0.00352159	0.000633723	0.00355425	0.00377237	0.000685785	0.00350304	0.000790552	0.000680639

Table 7: Objective function value and exact values of all parameters fitted via particle swarm in the parameter estimation starting from random start values. Parameter Sets 21 - 30 for the original model. The parameters are: complex association k_1 – 1, IκBα complex association $k4_2$ – 2, IκBα complex phosphorylation k_{cat} – 3, complex phosphorylation k_M – 4, complex shuttle k_1 – 5, complex shuttle k_2 – 6, IκBα degradation cyt k_1 – 7, IκBα degradation complex c k_1 – 8, IκBα degradation nuc complex k_1 – 9, IκBα degradation nuc k – 10, IκBα mRNA degradation cyt k – 11, IκBα mRNA degradation nuc k – 12, IκBα mRNA transport k – 13, IκBα p degradation k – 14, IκBα phosphorylation k_{cat} – 15, IκBα phosphorylation k_M – 16, IκBα nuclear shuttle k_1 – 17, IκBα nuclear shuttle k_2 – 18, IκBα basal transcription k – 19, IκBα transcription k_A – 20, IκBα translation k – 21, IKKβ active phosphorylation k_A – 22, IKKβ active phosphorylation k_M – 23, IKKβ active phosphorylation v_{max} – 24, IKKβ basal phosphorylation k – 25, IKKβ dephosphorylation k – 26, k IκBα-mRNA (scaling factor, scaling factor, experimental set1): – 27, k IκBα-mRNA (scaling factor, experimental set 2): – 28, k pIκBα (scaling factor, experimental set 1): – 29, k pIκBα (scaling factor, experimental set 2): – 30, k pIκBα (scaling factor, experimental set 3): – 31, k pIκBα (scaling factor, experimental set4): – 32, k IκBα (scaling factor, experimental set 1): – 33, k IκBα (scaling factor, experimental set 2): – 34, k IκBα (scaling factor, experimental set 3): – 35, k IκBα (scaling factor, experimental set 4): – 36, k pp65 (scaling factor, experimental set 1): – 37, k pp65 (scaling factor, experimental set 2): – 38, k pp65 (scaling factor, experimental set 3): – 39, k pp65 (scaling factor, experimental set 4): – 40, p65 degradation k1 – 41, p65 shuttle k1 – 42, p65 shuttle k2 – 43, p65 translation k – 44, TNFα degradation k1 – 45.

Parameter \ Fit	1	2	3	4	5	6	7	8	9	10
Obj. Fun. Value:	15153	16161.4	15545.3	15079.1	15778.2	15507.2	15771.6	15487.4	15815.1	15475.6
1	7.56098	0.975027	2.43463	7.5591	7.5591	4.7954	7.55428	7.55428	0.724117	3.77491
2	0.00367361	2.15452E-006	0.00214503	0.00390407	0.00390407	0.000395752	0.00021988	0.00021988	0.000001357	0.00169694
3	0.0012615	0.0125738	0.0010649	0.0010507	0.0010507	0.00122856	0.000135899	0.000135899	0.00234181	0.00182234
4	5.84068E-005	6.15842E-007	5.85071E-005	5.77825E-005	5.77825E-005	0.000000677	5.91354E-007	5.91354E-007	8.4629E-007	0.000000744
5	48.0728	605.626	60.3091	3054.47	3054.47	4437.54	588.353	588.353	49.6946	1668.66
6	0.624842	0.708045	0.0626759	6.23554	6.23554	6.06667	6.24873	6.24873	6.0798	0.0643103
7	1.78387E-007	2.33798E-007	1.78422E-007	2.49086E-007	2.49086E-007	1.78576E-007	1.9859E-007	1.9859E-007	1.80661E-007	1.93408E-007
8	2.99937E-005	0.000013768	1.69838E-007	2.85156E-007	2.85156E-007	2.14565E-007	1.70414E-007	1.70414E-007	8.59422E-007	1.47979E-005
9	1.59715E-007	1.11408E-007	1.11412E-007	1.11408E-007	1.11408E-007	1.13219E-007	0.000324869	0.000324869	0.000000427	6.07778E-007
10	6.97044E-006	4.96132E-005	0.000056703	3.85219E-006	3.85219E-006	1.32734E-006	1.67115E-006	1.67115E-006	1.44815E-006	5.82354E-006
11	0.00174965	0.00101467	0.000607034	0.000460559	0.000460559	0.00119339	0.000813514	0.000813514	0.00100948	0.002001
12	0.00106058	4.42004E-005	7.01101E-005	1.88081E-005	1.88081E-005	1.09765E-005	1.09261E-005	1.09261E-005	1.38312E-005	3.73719E-005
13	0.00669327	0.00669364	0.00685376	0.00669453	0.00669453	0.668577	0.00668831	0.00668831	0.588378	0.00670532
14	1.23615	3.80297	0.222827	1.13931	1.13931	13.1941	0.877073	0.877073	0.187594	5.48196
15	15.2897	99.9315	6.84153	19.3702	19.3702	81.8981	25.2323	25.2323	1.77151	127.52
16	0.00693951	0.00888787	0.00702838	0.00824702	0.00824702	0.0054374	0.0066514	0.0066514	0.0041897	0.00444955
17	12.8874	2.27819	14.0489	2.46947	2.46947	2.25677	4.1193	4.1193	10.9024	4.18198
18	218.563	276.699	663.321	149.676	149.676	196.512	149.545	149.545	887.163	513.171
19	1.79384	0.314252	10.6959	10.0891	10.0891	6.52973	7.63625	7.63625	0.214978	1.1715
20	0.748889	7.22208	1.71943	6.52104	6.52104	5.71979	5.36274	5.36274	12.3086	1.14672
21	0.0625166	0.0652393	0.0624634	0.0624634	0.0624634	0.0671002	0.0635603	0.0635603	0.0695933	0.063433
22	0.00204205	0.00426364	0.00419971	0.00502435	0.00502435	0.00179381	0.00221198	0.00221198	0.00281905	0.00176658
23	1.34884E-005	4.76728E-007	2.01116E-006	4.37286E-007	4.37286E-007	9.8383E-007	1.65077E-006	1.65077E-006	4.19229E-007	1.11701E-005
24	91153.5	26393.7	7685.39	94168	94168	63189.9	16065.3	16065.3	37327.5	45631
25	0.00723956	7.37937E-005	0.000223846	7.39112E-005	7.39112E-005	0.000898544	0.000180063	0.000180063	9.36776E-005	0.00173697
26	1.15167	1.77825	0.273139	3.73286	3.73286	4.35646	0.497224	0.497224	3.1035	5.71192
27	2.68183E-006	7.95385E-005	7.7949E-006	1.09365E-005	1.09365E-005	5.68748E-005	2.59088E-005	2.59088E-005	6.91248E-005	0.000128262
28	0.000307712	0.00124688	0.000337307	0.00031856	0.00031856	0.00141425	0.00130161	0.00130161	0.0014625	0.00219933
29	0.0559579	0.00316334	0.00631363	0.00194476	0.00194476	0.00900038	0.0126142	0.0126142	0.00258409	0.0531018
30	0.0559919	0.00374478	0.00908242	0.00251226	0.00251226	0.0110486	0.016428	0.016428	0.00341335	0.0554411
31	1178.53	1181.07	488.061	762.16	762.16	1038.78	1327.01	1327.01	542.796	519.389
32	773.757	763.992	321.488	503.801	503.801	680.777	874.639	874.639	354.343	340.6
33	925.163	910.264	383.532	604.173	604.173	818.711	1046.05	1046.05	425.207	408.451
34	701.263	677.944	285.947	459.509	459.509	619.455	789.002	789.002	320.231	308.788
35	157.451	125.468	62.1958	86.0839	86.0839	171.573	173.466	173.466	128.82	105.928
36	150.055	120.79	58.808	82.2675	82.2675	163.567	166.604	166.604	123.041	101.226
37	158.611	128.691	62.3158	86.8639	86.8639	172.995	173.667	173.667	130.722	107.101
38	166.015	136.433	65.1621	90.5978	90.5978	180.197	178.796	178.796	136.398	111.497

39	1719.65	771.31	2226.04	1475.14	1475.14	765.413	2518.9	2518.9	380.014	901.099
40	1957.06	878.223	2548.34	1691.87	1691.87	870.265	2856.66	2856.66	436.382	1027.43
41	2262.3	1022.77	2941.76	1947.78	1947.78	1008.26	3310.71	3310.71	508.09	1186.16
42	1758.6	790.67	2288.63	1531.41	1531.41	783.992	2594.44	2594.44	392.653	922.163
43	0.953121	79.2038	13.1646	39.1654	39.1654	17.774	89.3859	89.3859	3.389	91.0933
44	4732.1	10793.8	454356	9843.31	9843.31	7305.69	121216	121216	4594.73	17952.6
45	1.85323	0.0281895	0.0279505	1.07304	1.07304	0.0617926	0.0288867	0.0288867	0.0597794	2.7644
46	0.0119473	0.0327195	0.00705598	0.0135732	0.0135732	0.161078	0.0800172	0.0800172	0.030396	0.124747
47	0.0117961	0.0877289	0.0836277	0.0114108	0.0114108	0.0890865	0.088191	0.088191	0.0876779	0.0844156
48	205.104	192.711	18.183	200.527	200.527	76.1639	24.6898	24.6898	21.1039	47.1734
49	0.109424	0.0999892	0.0939508	0.843807	0.843807	6.39629	0.0951657	0.0951657	0.0935347	0.661259
50	0.00226192	2.57498E-005	0.00225915	0.000700716	0.000700716	0.000387792	0.00033952	0.00033952	0.000363064	0.000457522
51	2.02911E-006	1.59157E-005	9.06243E-007	2.66486E-005	2.66486E-005	1.25009E-005	3.95211E-005	3.95211E-005	3.90966E-005	1.71683E-005
52	0.000251458	0.000258247	0.0251042	0.000254016	0.000254016	0.000289324	0.000251126	0.000251126	0.000401172	0.000260995
53	79.9937	79.7133	0.800015	79.9717	79.9717	0.847462	79.7792	79.7792	77.1219	79.5527
54	0.0359883	0.0375709	0.0930566	0.0949199	0.0949199	0.0326323	0.131996	0.131996	0.0289167	0.114842
55	6.31764E-005	8.14075E-006	0.000809925	6.89947E-005	6.89947E-005	0.000174277	0.000808072	0.000808072	8.46941E-006	0.000567295
56	2.06146E-007	2.31727E-006	1.7831E-007	4.99369E-006	4.99369E-006	1.23123E-006	2.91982E-006	2.91982E-006	0.000005284	2.04451E-006
57	0.00822442	0.0023847	0.0188307	0.0147883	0.0147883	0.000789359	0.00401138	0.00401138	0.00138974	0.00321804
58	6596.53	6775.62	189.092	275.061	275.061	5071.39	1603.61	1603.61	160.217	125.089
59	0.796647	0.806405	0.00828226	0.807252	0.807252	0.247144	0.807243	0.807243	0.795006	0.293453
60	0.00185192	0.00231532	0.00160006	0.00185296	0.00185296	0.00225113	0.00249447	0.00249447	0.00552428	0.00440004
61	0.00172071	0.000389915	0.00176522	0.00136116	0.00136116	0.000333962	0.000386861	0.000386861	0.000378729	0.000296833

Table 8: Objective function value and exact values of all parameters fitted via particle swarm in the parameter estimation starting from random start values. Parameter Sets 1 - 10 for the expanded model. The parameters are: 1 – complex association k_1, 2 – complex association k_2, 3 – complex nuclear shuttle k_1, 4 – complex nuclear shuttle k_2, 5 – IκBα complex phosphorylation k_cat, 6 – IκBα complex phosphorylation k_M, 7 – IκBα deg complex cyt k, 8 – IκBα deg complex nuc k, 9 – IκBα deg cyt k, 10 – IκBα deg nuc k, 11 – IκBα-mRNA deg cyt k, 12 – IκBα-mRNA deg nuc k, 13 – IκBα-mRNA transport k, 14 – IκBα nuclear shuttle k_1, 15 – IκBα nuclear shuttle k_2, 16 – IκBα p active degradation k, 17 – IκBα phosphorylation k_{cat}, 18 – IκBα phosphorylation k_M, 19 – IκBα transcription k_A1 pSer536p65, 20 – IκBα transcription k_A2 p65, 21 – IκBα transcription k_A3 pSer276p65, 22 – IκBα basal transcription k, 23 – IκBα translation k, 24 – IKKβ active phosphorylation k_A, 25 – IKKβ active phosphorylation k_M, 26 – IKKβ active phosphorylation v_{max}, 27 – IKKβ basal phosphoylation k, 28 – IKKβ dephopshorylation k, 29 – MSK1 activation k_{cat}, 30 – MSK1 activation k_M, 31 – MSK1 deactivation, 32 – MSK1 signal depletion k, 33 – MSK1 signal transmission 2 k, 34 – MSK1 signal transmission kA, 35 – MSK1 signal transmission k_M, 36 – MSK1 signal transmission v_max, 37 – p65 degradation k, 38 – p65 MSK1 phosphorylation k_M, 39 – p65 MSK1 phosphrylation k_{cat}, 40 – p65 nuclear shuttle k_1, 41 – p65 nuclear shuttle k_2, 42 – p65 translation k, 43 – pSer536p65 dephosphorylation k, 44 – pp65 MSK1 phosphrylation k_{cat}, 45 – pp65 MSK1 phosphrylation k_M, 46 – pSer276p65 dephosphorylation, 47 – TNFα amount, 48 – TNFα degradation k, 49 – turnover IκBα, 50 – turnover p65.

Parameter \ Fit	11	12	13	14	15	16	17	18	19	20
Obj. Fun. Value:	15924.2	15639	15852.7	15631.9	15825.3	15477.3	15204.4	15204.4	15164.6	15460.2
1	2.72169	7.54676	1.62334	7.0099	7.2877	7.55998	7.56009	7.56097	2.45918	1.94513
2	3.08963E-005	0.00419888	1.01461E-006	0.00309984	0.0116346	0.00263824	0.00734051	0.00383838	0.00194324	7.24552E-005
3	0.00113557	0.00126626	0.000999305	0.00134667	0.00187062	0.00141446	0.00165165	0.00102733	0.00181346	0.00124151
4	5.82715E-005	3.29117E-006	9.36502E-007	0.000004052	1.01274E-006	0.000057728	5.1519E-006	5.85237E-007	2.99611E-005	5.85204E-007
5	4442.02	1313.43	131.238	337.479	4769.62	1416.03	312.929	48.1842	53.2153	125.548
6	0.494298	5.07875	1.74882	2.22699	0.0820852	0.570582	5.99078	6.26549	0.0816325	6.26749
7	0.000000209	3.23086E-007	1.83756E-007	1.90763E-007	1.83523E-007	1.78094E-007	3.81548E-007	1.93401E-007	1.79528E-007	2.30052E-007
8	2.99937E-005	4.63252E-007	2.98458E-005	1.69826E-007	1.91146E-005	2.28367E-007	4.01629E-007	2.69776E-005	2.99981E-005	2.22084E-005
9	1.59932E-007	2.89327E-007	2.30326E-007	1.11422E-007	1.11598E-007	1.54598E-007	1.71399E-007	1.27539E-007	3.08571E-007	1.81291E-007
10	3.92056E-006	4.67944E-006	1.56837E-006	6.94505E-006	1.3539E-006	1.55812E-006	3.78019E-006	1.94383E-006	0.000710322	1.30648E-006
11	0.0027022	0.00125919	0.00225705	0.000778207	0.00181743	0.000578612	0.00162511	0.000446143	0.000543185	0.0019982
12	0.000414381	0.000329963	5.49381E-005	1.82921E-005	1.58347E-005	1.57283E-005	1.26028E-005	7.22038E-005	0.000959414	0.000708651
13	0.0066862	0.376167	0.633024	0.354806	0.00669015	0.00670123	0.00668637	0.00676062	0.0287777	0.0067063
14	0.262849	20.9521	72.7001	2.90397	39.9037	43.2815	129.329	2.82458	15.2414	19.9638
15	3.94565	264.073	493.599	38.4059	670.045	347.251	1195.82	45.611	237.016	138.825
16	0.0119243	0.00474002	0.0107295	0.00554793	0.00579091	0.00965086	0.00366727	0.0105378	0.00630775	0.0060178
17	14.5614	8.76056	6.81111	7.36999	25.6681	11.1916	25.913	1.66481	10.3619	9.73439
18	878.135	461.684	343.146	111.736	447.503	95.9667	860.812	357.071	366.175	258.123
19	15.6736	14.8349	4.45615	4.87431	3.99346	5.261	0.586707	12.6889	8.94255	2.77783
20	4.54709	11.348	2.22058	3.51604	2.60121	4.37802	1.02467	6.82699	3.32073	3.38407
21	0.0629058	0.340142	0.0624703	0.0633614	0.0648504	0.0626783	0.0735502	0.062469	0.062469	0.0630114
22	0.00151708	0.00176001	0.00136081	0.00242008	0.00179394	0.00337699	0.00194337	0.00498841	0.00642438	0.00156865
23	3.31325E-006	8.61665E-007	5.74277E-006	1.13244E-006	3.52871E-006	6.7245E-007	8.84932E-006	3.60513E-007	1.01384E-006	6.95277E-006
24	125425	38629.9	220241	58028.6	202603	215152	127321	87914.5	92766.9	240221
25	0.00724725	0.000120024	0.000754961	0.000710346	7.59565E-005	0.00129538	0.00261276	7.25748E-005	7.75602E-005	0.000304043
26	4.03473	1.96477	6.11121	0.760125	3.09202	0.971426	5.57212	10.5665	2.64268	4.85466
27	3.28265E-005	4.47957E-005	2.68773E-005	0.000012682	1.52291E-005	0.000004858	3.28367E-005	4.44212E-005	7.88973E-006	1.98673E-005
28	0.00132958	0.00190144	0.00128804	0.00182707	0.00207748	0.00118386	0.00238937	0.000312055	0.000377531	0.00165038
29	0.0218902	0.0076748	0.0366489	0.012135	0.0211862	0.00586517	0.055951	0.00126739	0.00808049	0.0559373
30	0.0265829	0.0105198	0.0486945	0.0149292	0.0289789	0.00780578	0.055896	0.00177615	0.00976059	0.0559915
31	1612.42	918.771	1428.17	1139.8	735.84	1694.44	1292.94	818.401	1059.13	823.71
32	1077.17	612.08	944.416	759.561	487.339	1099.51	847.643	540.587	693.57	542.263
33	1272.66	729.815	1126.89	909.924	579.292	1312.76	1028.57	645.563	821.441	649.828
34	951.635	545.527	849.097	683.151	436.494	994.391	762.303	491.222	621.48	490.199
35	114.278	173.356	112.409	184.868	114.224	150.457	312.515	72.564	161.734	120.592
36	109.859	165.826	107.63	178.834	109.615	143.556	299.926	69.2913	155.772	115.743
37	116.403	177.355	113.73	188.705	116.64	152.376	319.37	73.2556	164.209	122.362
38	120.849	182.093	117.662	196.088	121.378	159.187	330.344	75.7755	171.338	127.193

39	2025.62	2204.28	1212.14	1445.45	1224.29	1729.51	2020.01	1396.76	1827.64	1092.12
40	2319.29	2498.37	1378.97	1673.58	1395.59	1990.12	2303.81	1603.08	2078.88	1241.75
41	2652.7	2873.54	1591.18	1908.87	1605.17	2284.27	2671.07	1835.28	2397.88	1429.51
42	2079.5	2249.74	1244.48	1502.01	1260.5	1792.59	2093.25	1451.66	1884.19	1130.39
43	64.1272	89.3751	5.22734	49.6733	0.912086	22.1764	5.06812	91.1963	5.00513	41.4097
44	216912	216002	133450	446467	454254	4543.76	309031	452165	4551.03	101476
45	2.59834	2.05737	2.78369	0.193111	2.5728	0.134228	0.0293114	2.74023	2.7882	2.79494
46	0.102366	0.0751754	0.0377524	0.0310693	0.0315368	0.107841	0.00286767	0.00444237	0.00788904	0.214432
47	0.0891471	0.00682785	0.0795974	0.086974	0.0620172	0.0851729	0.0631418	0.0891644	0.0860888	0.0891645
48	54.8654	23.5573	71.7129	81.5552	10.3851	87.5108	24.4833	23.8907	86.542	44.0442
49	0.0937126	0.306994	3.95491	1.42185	8.84431	0.0970505	0.0941387	0.0935354	0.0935741	0.0935347
50	0.000475488	0.00103317	0.00226191	0.00205721	0.00225412	0.00226189	3.76044E-005	0.000877594	0.00122525	0.00222736
51	3.58309E-005	3.93183E-005	3.66104E-005	4.99703E-005	1.32891E-005	5.00388E-005	0.000043631	5.00407E-005	1.28944E-005	2.05279E-005
52	0.0243666	0.000261038	0.000253001	0.000346237	0.000263631	0.000251158	0.0250363	0.000251125	0.0250985	0.000251288
53	79.346	70.5463	79.7593	79.9873	22.6788	79.9531	0.8	79.7885	79.1481	51.8996
54	0.105346	0.0901822	0.0561801	0.0589067	0.0531242	0.04555	0.0354069	0.0848503	0.025157	0.0852691
55	3.58165E-005	0.000649955	8.0998E-006	0.000281474	1.38004E-005	3.32283E-005	8.10016E-006	0.000008222	1.17411E-005	8.41573E-006
56	4.78307E-006	0	5.19081E-006	0.000003787	1.42244E-006	6.25578E-006	1.50928E-006	1.23163E-005	1.07341E-006	2.36851E-006
57	0.00416911	0.00564142	0.00258707	0.00386022	0.00825922	0.0303455	0.00397285	0.0282963	0.00683475	0.00204034
58	11855.2	246.199	10818	2463.83	11787.2	119.31	590.552	119.27	1773.05	695.631
59	0.376466	0.347728	0.113781	0.746966	0.117612	0.807145	0.18222	0.806085	0.801639	0.239715
60	0.00257116	0.0055523	0.00245739	0.00257059	0.00294369	0.0147379	0.00382412	0.00409829	0.00471701	0.0012094
61	0.000312749	0.00031087	0.000313922	0.000313049	0.000290272	0.000329454	0.00030796	0.00110797	0.00175347	0.000332961

Table 9: Objective function value and exact values of all parameters fitted via particle swarm in the parameter estimation starting from random start values. Parameter Sets 11 - 20 for the expanded model. The parameters are: 1 – complex association k_1, 2 – complex association k_2, 3 – complex nuclear shuttle k_1, 4 – complex nuclear shuttle k_2, 5 – IκBα complex phosphorylation k_cat, 6 – IκBα complex phosphorylation k_M, 7 – IκBα deg complex cyt k, 8 – IκBα deg complex nuc k, 9 – IκBα deg cyt k, 10 – IκBα deg nuc k, 11 – IκBα-mRNA deg cyt k, 12 – IκBα-mRNA deg nuc k, 13 – IκBα-mRNA transport k, 14 – IκBα nuclear shuttle k_1, 15 – IκBα nuclear shuttle k_2, 16 – IκBα p active degradation k, 17 – IκBα phosphorylation k_{cat}, 18 – IκBα phosphorylation k_M, 19 – IκBα transcription k_A1 pSer536p65, 20 – IκBα transcription k_A2 p65, 21 – IκBα transcription k_A3 pSer276p65, 22 – IκBα basal transcription k, 23 – IκBα translation k, 24 – IKKβ active phosphorylation k_A, 25 – IKKβ active phosphorylation k_M, 26 – IKKβ active phosphorylation v_{max}, 27 – IKKβ basal phosphoylation k, 28 – IKKβ dephopshorylation k, 29 – MSK1 activation k_{cat}, 30 – MSK1 activation k_M, 31 – MSK1 deactivation, 32 – MSK1 signal depletion k, 33 – MSK1 signal transmission 2 k, 34 – MSK1 signal transmission kA, 35 – MSK1 signal transmission k_M, 36 – MSK1 signal transmission v_max, 37 – p65 degradation k, 38 – p65 MSK1 phosphorylation k_M, 39 – p65 MSK1 phosphrylation k_{cat}, 40 – p65 nuclear shuttle k_1, 41 – p65 nuclear shuttle k_2, 42 – p65 translation k, 43 – pSer536p65 dephosphorylation k, 44 – pp65 MSK1 phosphrylation k_{cat}, 45 – pp65 MSK1 phosphrylation k_M, 46 – pSer276p65 dephosphorylation, 47 – TNFα amount, 48 – TNFα degradation k, 49 – turnover IκBα, 50 – turnover p65.

Parameter \ Fit	21	22	23	24	25	26	27	28	29	30
Obj. Fun. Value:	15642.4	15930.9	16051.2	16419.5	15370.1	17951.7	15588.2	15579.7	18930.4	16440.6
1	3.94998	3.2452	5.53324	0.588924	7.56098	1.48249	6.89118	3.70751	7.0445	1.19539
2	0.00220364	0.00516698	0.00144061	1.04108E-006	0.00280335	0.00103531	0.000215364	0.00172246	0.000001	1.00326E-006
3	0.000900496	0.00229555	0.00164418	0.00165528	0.000982837	0.0025608	0.000272448	0.000995919	0.0020616	0.0135705
4	6.27729E-007	5.60363E-005	4.16378E-006	6.71836E-007	5.84769E-005	1.49863E-005	5.85336E-007	5.74183E-005	5.85684E-007	1.1275E-006
5	284.034	49.7075	2420.57	4801.19	48.0304	49.0857	2160.83	97.7627	48.6036	1705.94
6	0.0627015	0.0850825	0.943953	3.00806	0.0734829	3.46239	0.675707	0.48111	6.20775	2.88035
7	2.56246E-007	1.85653E-007	7.94516E-007	2.15193E-007	3.12727E-007	1.31747E-006	2.01309E-007	1.97459E-007	2.99982E-005	2.46947E-007
8	0.000018475	2.91667E-005	8.11429E-006	2.45684E-007	1.69938E-007	2.99727E-005	1.25669E-005	0.000028897	2.68926E-005	4.27067E-007
9	3.63529E-006	2.51231E-007	1.26157E-007	0.000000125	5.97682E-007	1.19434E-007	0.000782402	3.75879E-007	1.12672E-007	1.12141E-007
10	1.37528E-006	2.86313E-006	4.00394E-005	1.11527E-005	0.00499587	0.00103843	1.30109E-006	1.5689E-006	1.30042E-006	1.30042E-006
11	0.00581426	0.000689314	0.000708997	0.000451284	0.00199201	0.00290599	0.00215492	0.000674345	0.000303687	0.00286541
12	0.0010921	0.000018932	0.000535145	2.66407E-005	2.71623E-005	0.00056383	1.46713E-005	1.09232E-005	0.00109219	0.000571516
13	0.00839912	0.668428	0.00672518	0.00668594	0.00669104	0.00669972	0.653791	0.00668594	0.0202126	0.00670092
14	129.013	41.7059	2.22574	49.3221	24.5501	14.741	4.38583	13.9973	16.2289	0.535395
15	13.8294	570.262	233.109	242.223	94.5261	1173.48	64.2687	222.578	337.046	33.8282
16	0.00451566	0.0048617	0.00785661	0.0184579	0.0120102	0.0234313	0.0097815	0.0109104	0.00531453	0.00660883
17	0.815567	11.9072	10.3567	22.7904	7.20099	14.8458	12.4454	0.386097	4.25985	10.2342
18	114.777	314.32	582.223	756.053	71.9482	391.038	878.139	98.9485	65.1958	584.479
19	13.655	1.58173	1.5741	0.844471	5.48061	6.18357	0.221058	5.78124	21.4743	0.216697
20	3.2535	1.4818	0.721115	1.23734	1.86567	0.808142	4.87687	2.16131	0.123129	1.33
21	5.26538	2.04821	4.86635	0.0636605	0.0624637	0.127627	0.0624782	0.0624678	5.3494	0.0624677
22	0.000925017	0.00303161	0.00311963	0.00427673	0.00212897	0.00179401	0.00140066	0.00293018	0.00661559	0.00236633
23	8.21932E-006	2.00528E-006	4.24183E-006	1.43672E-006	6.41354E-006	2.39919E-005	6.01584E-006	1.69733E-006	2.10539E-007	5.94754E-006
24	15345.2	57103.7	171882	93474.9	153595	156233	173013	74510.6	310882	143049
25	0.00059754	0.000096921	0.00700532	0.00725227	7.36865E-005	0.000422549	0.000074015	7.55377E-005	0.000125368	0.0072554
26	2.54698	1.52461	6.85719	1.56869	0.857285	2.98841	8.93036	10.3437	3.81356	5.97143
27	3.13644E-005	2.35422E-005	4.13981E-005	0.000019543	2.31911E-006	2.88206E-005	3.89133E-005	0.000127506	1.07825E-005	4.76483E-005
28	0.000300782	0.0022078	0.00172132	0.00129824	0.000403305	0.00155095	0.000724036	0.00081124	0.00141276	0.00183027
29	0.0549041	0.0186684	0.018292	0.00643993	0.0260272	0.000580848	0.0436559	0.00535479	0.00166421	0.0398821
30	0.0553553	0.0248211	0.0240467	0.0080997	0.0319876	0.0519435	0.0559841	0.00624366	0.00204877	0.0450794
31	709.73	946.471	711.354	1675.74	964.784	1253.12	1990.79	664.213	827.589	914.824
32	465.782	624.038	473.322	1109.96	636.322	804.903	1293.49	438.587	540.859	595.95
33	563.102	755.141	565.3	1300.73	760.626	923.04	1531.85	524.605	649.982	713.157
34	431.407	560.14	419.186	976.154	575.194	685.406	1159.04	397.404	489.893	528.237
35	166.904	178.99	81.4407	81.5782	79.128	63.2142	213.682	54.6842	140.052	128.854
36	158.64	171.668	77.6505	78.1091	75.8459	61.0275	205.255	52.567	134.43	123.542
37	168.902	183.159	82.4004	82.7184	79.646	63.3347	215.405	55.6932	142.215	130.325
38	176.54	189.668	86.2144	86.6857	83.1122	63.6334	221.667	58.0414	142.884	135.448

39	567.796	1234.35	1541.99	505.663	637.894	2272.78	1270.49	1007.51	398.445	839.389
40	656.045	1410.67	1762.92	577.212	719.372	2617.34	1436.13	1152.74	461.708	961.186
41	759.781	1613.83	2033.01	672.692	839.095	2978.84	1661.13	1322.38	527.352	1112.66
42	584.055	1269.13	1588.65	515.651	653.425	2338.23	1301.63	1037.91	416.661	868.719
43	56.47	46.247	9.12722	8.4296	4.95352	27.7777	88.9417	1.82568	91.1639	90.8479
44	13295.5	4595.36	80077.7	11943.2	51337.8	100646	66371.1	4543.77	4572.9	35173.9
45	0.0854885	2.75162	0.140076	2.51529	0.084671	0.604208	0.0308139	1.57484	2.7949	0.426761
46	0.00360968	0.247413	0.0163225	0.0317313	0.00486216	0.0114418	0.0624146	0.0105813	0.0224583	0.103162
47	0.0807393	0.0761844	0.0879904	0.089161	0.0823482	0.0651016	0.0891119	0.0886135	0.00179631	0.089065
48	207.728	385.555	119.895	206.212	21.3271	99.8219	9.03478	72.6816	106.062	84.855
49	0.72175	0.10133	4.71717	0.109257	0.0935477	0.131581	0.940253	0.0968009	0.0936955	8.53057
50	0.00170177	0.000363636	0.0022591	0.000931228	0.000835574	0.000401123	0.00226148	0.00225938	0.00220756	0.00206726
51	5.01306E-006	3.10975E-005	2.12231E-005	3.05854E-005	1.29557E-005	2.25609E-005	2.51051E-005	1.36576E-005	6.61678E-006	5.00283E-005
52	0.00144817	0.00471382	0.0137962	0.000275936	0.00025122	0.0250123	0.000251126	0.000251995	0.00299521	0.000251261
53	36.2932	0.877777	1.04473	79.9705	75.4103	11.8465	23.8702	79.9573	79.9959	79.8371
54	0.00694719	0.0279425	0.282762	0.0306772	0.0274935	0.125786	0.0214664	0.112766	0.00925509	0.0731953
55	0.000808469	8.24383E-006	8.26314E-006	8.45432E-006	8.2356E-006	9.08748E-006	8.56392E-006	9.12832E-006	1.40516E-005	9.49612E-006
56	4.70268E-007	2.19533E-006	3.37473E-006	0.000007407	3.09662E-006	3.81607E-006	2.11305E-006	4.22269E-006	2.88719E-006	6.72459E-006
57	0.00302465	0.00520257	0.00819773	0.00518256	0.00650751	0.013769	0.00397842	0.0141051	0.0143802	0.00238636
58	2101.07	1527.95	4902.45	195.207	650.593	182.975	119.258	191.779	6047.21	904.285
59	0.00867793	0.596571	0.703362	0.00817367	0.254238	0.00842206	0.805476	0.807248	0.00807297	0.412642
60	0.00676548	0.0273317	0.120393	0.00552562	0.00254724	0.00332831	0.00622356	0.00550202	0.112692	0.120835
61	0.000922099	0.000294254	0.000317609	0.000307332	0.000834357	0.00044076	0.000556895	0.000393283	0.000378464	0.000349378

Table 10: Objective function value and exact values of all parameters fitted via particle swarm in the parameter estimation starting from random start values. Parameter Sets 21 - 30 for the expanded model. The parameters are: 1 – complex association k_1, 2 – complex association k_2, 3 – complex nuclear shuttle k_1, 4 – complex nuclear shuttle k_2, 5 – IκBα complex phosphorylation k_cat, 6 – IκBα complex phosphorylation k_M, 7 – IκBα deg complex cyt k, 8 – IκBα deg complex nuc k, 9 – IκBα deg cyt k, 10 – IκBα deg nuc k, 11 – IκBα-mRNA deg cyt k, 12 – IκBα-mRNA deg nuc k, 13 – IκBα-mRNA transport k, 14 – IκBα nuclear shuttle k_1, 15 – IκBα nuclear shuttle k_2, 16 – IκBα p active degradation k, 17 – IκBα phosphorylation k_{cat}, 18 – IκBα phosphorylation k_M, 19 – IκBα transcription k_A1 pSer536p65, 20 – IκBα transcription k_A2 p65, 21 – IκBα transcription k_A3 pSer276p65, 22 – IκBα basal transcription k, 23 – IκBα translation k, 24 – IKKβ active phosphorylation k_A, 25 – IKKβ active phosphorylation k_M, 26 – IKKβ active phosphorylation v_{max}, 27 – IKKβ basal phosphoylation k, 28 – IKKβ dephopshorylation k, 29 – MSK1 activation k_{cat}, 30 – MSK1 activation k_M, 31 – MSK1 deactivation, 32 – MSK1 signal depletion k, 33 – MSK1 signal transmission 2 k, 34 – MSK1 signal transmission kA, 35 – MSK1 signal transmission k_M, 36 – MSK1 signal transmission v_max, 37 – p65 degradation k, 38 – p65 MSK1 phosphorylation k_M, 39 – p65 MSK1 phosphrylation k_{cat}, 40 – p65 nuclear shuttle k_1, 41 – p65 nuclear shuttle k_2, 42 – p65 translation k, 43 – pSer536p65 dephosphorylation k, 44 – pp65 MSK1 phosphrylation k_{cat}, 45 – pp65 MSK1 phosphrylation k_M, 46 – pSer276p65 dephosphorylation, 47 – TNFα amount, 48 – TNFα degradation k, 49 – turnover IκBα, 50 – turnover p65.

experimental set 1			
Time in min	phospho-p65	IκBα	phospho-IκBα
0	32.33	67.51	23.51
5	47.76	67.34	100
10	100	22.48	67.72
20	48.92	7.86	22.39
40	36.2	32.77	23.65
60	29.23	64.25	40.6
120	28.37	100	28.48
180	32.49	78.87	25.84
240	34.52	77.72	18.72
experimental set 2			
Time in min	phospho-p65	IκBα	phospho-IκBα
0	16.11	71.73	30.37
5	37.36	91.08	35.84
10	100	49.88	100
20	79.45	15.13	29.97
40	48.36	22.93	37.83
60	42.49	60.98	70.3
120	63.59	100	68.08
180	57.32	66.35	53.02
240	21.52	78.76	27.35
Experiment 3			
Time in min	phospho-p65	IκBα	phospho-IκBα
0	40.42	82.97	26.92
5	43.27	100	33.99
10	100	54.29	100
20	53.15	15.51	22.79
40	15	18.71	11.08
60	17.23	65.63	29.65
120	23.15	96.22	43.5
180	4.51	60.97	27.26
experimental set 4			
Time in min	phospho-p65	IκBα	phospho-IκBα
0	29.97	68.69	45.47
5	33.7	78.67	89.5
10	100	40.3	100
20	43.11	26.04	58.69
40	20.06	33.69	40.59
60	37.48	100	97.33
120	28.2	90.59	40.19
180	14.72	70.78	40.89
240	5.47	58.98	80.0

Table 11: Relative protein concentrations over time determined from immuno blotting.

experimental set 1	
Time in min	IκBα mRNA
0	1
5	1.24
10	1.1
20	1.14
40	4.76
60	6.49
120	3.88
180	4.56
experimental set 2	
Time in min	IκBα mRNA
0	1
5	0.92
10	0.91
20	1.15
40	4.43
60	4.98
120	2.95
180	2.73

Table 12: Relative mRNA concentrations over time determined from quantitative reverse transcription polymerase chain reaction.

References

[1] Louise Ashall, Caroline A Horton, David E Nelson, Pawel Paszek, Claire V Harper, Kate Sillitoe, Sheila Ryan, David G Spiller, John F Unitt, David S Broomhead, Douglas B Kell, David A Rand, Violaine Sée, and Michael R H White. Pulsatile stimulation determines timing and specificity of NF-κB-dependent transcription. *Science*, 324(5924):242–6, Apr 2009.

[2] Ellen L O'Dea, Derren Barken, Raechel Q Peralta, Kim T Tran, Shannon L Werner, Jeffrey D Kearns, Andre Levchenko, and Alexander Hoffmann. A homeostatic model of IκB metabolism to control constitutive NF-κB activity. *Mol Syst Biol*, 3:111, 2007.

[3] MJ de Hoon, S Imoto, J Nolan, and S Miyano. Open source clustering software. *Bioinformatics (Oxford, England)*, 20(9):1453–1454, 2004.
